# Sustainable closed-loop supply chain network design under uncertainty using a fuzzy multi-objective optimization framework for the battery industry

**DOI:** 10.1038/s41598-026-47477-8

**Published:** 2026-04-16

**Authors:** Mahdi Yousefi Nejad Attari, Sahar Rezanejad, Ali Ala, Vladimir Simic, Dragan Pamucar

**Affiliations:** 1 Department of Industrial Engineering, Bon.C.,, Islamic Azad University, Bonab, Iran; 2 Department of Mathematics, Saveetha School of Engineering, Saveetha Institute of Medical and Technical Sciences (SIMATS), Chennai, Tamil Nadu 602105 India; 3 School of Mechanical & Materials Engineering, University College Dublin, Dublin, D04 V1W8 Ireland; 4 Sustainability Competence Centre, Széchenyi István University, Egyetem Tér 1, Győr, 9026 Hungary; 5 College of Engineering, Department of Industrial Engineering and Management, Yuan Ze University, 320315 Taoyuan , Taiwan; 6 UNEC Applied Artificial Intelligence Research Center, Azerbaijan State University of Economics (UNEC), Baku, Azerbaijan

**Keywords:** Closed-loop supply chain, Sustainable, Optimization, Metaheuristic algorithms, Disruptions, Energy science and technology, Engineering, Mathematics and computing

## Abstract

The study presents a sustainable closed-loop supply chain network that integrates financial, environmental, and social objectives within a context of uncertainty. A fuzzy-based modeling approach is introduced to address uncertainty in customer demand, cost parameters, and carbon emission coefficients across the sustainable closed-loop supply chain network. Two metaheuristic methods, the non-dominated sorting genetic algorithm II (NSGA-II) and multi-objective particle swarm optimization (MOPSO), are employed to address the problem and are compared against each other. A practical case study of a battery company is employed to validate the framework. The findings indicate that MOPSO surpasses non-dominated sorting genetic algorithm II in terms of solution quality and computational efficiency, compared with NSGA-II, the proposed MOPSO achieved a 6.3% reduction in total cost and an 8.1% decrease in CO₂ emissions, while the social index reflecting recruitment and employee security increased by 12.5%. This study contributes a sustainable closed-loop supply chain network design model for the battery industry that together optimizes economic, environmental, and social objectives amid parameter uncertainty, and offers algorithmic evaluations of optimized multi-objective metaheuristics to achieve high-quality Pareto solutions.

**Supplementary Information:**

The online version contains supplementary material available at 10.1038/s41598-026-47477-8.

## Introduction

The adverse environmental impacts of industrialization and our modern lifestyle have become a global problem, and there is an increasing need for solutions to these phenomena. The battery industry offers a crucial and appropriate application for developing sustainable closed-loop supply chain networks (CLSC). The rapid growth of battery manufacturing, driven by green transportation, renewable energy storage, and electronic products, has contributed to resource constraints, hazardous waste production, and environmental pollution. These attributes render the battery business particularly conducive to examining integrated sustainability- and reliability-focused CLSC frameworks^1^. This study defines sustainability as the balanced progression of economic growth, environmental conservation, and social welfare in the long run. The suggested closed-loop supply chain framework enhances material circularity and diminishes dependence on virgin resources by systematically guiding recovery and recycling decisions, thereby reducing extended environmental impacts. Concurrently, an economic network architecture ensures operational continuity, while social performance metrics reflect employment and regional development. Collectively, these components provide supply chain structures that meet present demand while mitigating negative environmental and socioeconomic effects on future generations. Administration support and related policies have made much of what has been achieved regarding sustainability in developed countries possible^2^. In recent decades, CLSC has been integrated with the conventional forward supply chain (SC) due to increasing concerns regarding resource scarcity and environmental contamination^3^. In other words, sustainability measures in less developed countries are often perceived as requiring serious investment or increased costs, so many producers are reluctant to implement them^4,5^. Meeting customer demands with minimal costs and exceptional quality, delivered punctually, has consistently been a primary objective of supply chain management (SCM). Nonetheless, fluctuating market conditions and changing client requirements present obstacles for firms in achieving these goals while maintaining international competitiveness. Thus, the design and execution of SCs that integrate client requirements have emerged as a solution, establishing a sustainable competitive advantage for firms. One of a business’s most important quality elements is to avoid production interruptions and service disruptions, such as incomplete and delayed delivery of goods/services. However, based on the nature and diversity of equipment, SC may have different types of failures^6^. Failure of one of the components of the SC network may disrupt the performance of the entire SC or, at best, reduce the efficiency of the chain. Therefore, considering the reliability factor in the design of CLSCs, especially in sustainable supply chains (SSCs), seems essential. As a result, an expanding delegation of investigators and practitioners is embracing SSC management, which is defined as the management of goods, data, and financial flows, accompanied by collaboration among enterprises within SC. Sustainable supply chain management (SSCM) incorporates objectives from the three dimensions of sustainable development: financial, environmental, and social, as demanded by client and stakeholder standards^7^.

On the one hand, researchers have recently turned to other extensions, such as reliability in designing SSCs. Coello et al^8^. proposed a multi-objective, multi-product, multi-period, and multi-modal supply chain model, whereby the nonlinear characteristics of the objective functions and constraints markedly boosted the computational complexity and solution time. Recent studies examine sustainable CLSC modeling utilizing optimization strategies that integrate reliability-related performance metrics with environmental and economic objectives^9^ suggesting that this research domain is expanding rather than being characterized by a singular study^10^. Also, in some other works the exact probabilistic distributions of uncertain variables required for the probabilistic programming approach are rarely available in behavior, necessitating dependence on approximation and assumption methodologies^11,12^.

Supply chain network design issues are fundamentally influenced by various sources of uncertainty, including volatile demand, variable costs, and unpredictable environmental and societal factors. Fuzzy programming is an effective method for modeling uncertainty when parameters cannot be accurately defined and are represented by linguistic phrases or imprecise numerical values via membership functions, making it especially well-suited to addressing epistemic doubt. Robust optimization seeks to guarantee solution feasibility and performance consistency notwithstanding unfavorable parameter fluctuations. Instead of relying on precise probability distributions, it aims for solutions that are effective across multiple plausible scenarios, thereby reducing susceptibility to uncertainty and enhancing decision reliability^13^.

This study integrates fuzzy programming and robust optimization to leverage their complementary advantages: fuzzy programming addresses parameter imprecision, whereas robust optimization improves solution stability. This comprehensive methodology facilitates a more realistic, resilient design of a closed-loop supply chain network amid unpredictability. Consequently, it is essential to implement a robust fuzzy optimization method to address the parameter uncertainty issue in the design of sustainable CLSC networks^14^.

This paper investigates a multi-tier, multi-objective, robust fuzzy network design problem involving plants, distribution centers, customers, collecting facilities, and manufacturing centers under uncertainty. This study seeks to identify optimal strategic options for SC stakeholders, focusing on cost reduction, enhancement of client satisfaction, and the planning of forward and reverse product flows inside the CLSC networks.

The contributions and novelties of this study compared to prior research are as follows:

(i) Developing and optimizing a robust fuzzy multi-objective framework tailored to the battery industry in the CLSC network.
(ii) Considering a hybrid metaheuristic solution strategy with Taguchi-optimized parameters to tackle medium- to large-scale problems.
(iii) Addressing comprehensive evaluation metrics and comparative investigation demonstrating the superiority of the presented method.
(iv) Analyzing a real case study problem to evaluate environmental and managerial insights.

The remainder of our paper is organized as follows: Section "Related Works" reviews relevant previous research. Section "Problem Description" introduces the problem description, assumptions, as well as background of research methodology, and the formulated multi-objective sustainable CLSC optimization framework. Section "Solution Approaches" gives solution approaches, including the multi-objective particle swarm optimization (MOPSO), as well as the non-dominated sorting genetic algorithm II (NSGA-II). Section "Case Study and Experimental Results" presents a case study, followed by a discussion of the numerical outcomes. Section "Conclusion" provides the principal conclusions and suggestions for future research.

## Related works

Traditional forward SCs typically involve processes such as raw material procurement, product manufacturing, transportation, inventory control, and distribution. While the forward flow primarily aims to reduce operational costs, the reverse flow emphasizes environmental sustainability by focusing on factors like component reliability, recoverability, and disassembly efficiency.

### Sustainable closed-loop supply chain network design

In this context, Goodarzian et al^15^. examined and solved the CLSC model. They presented a cumulative logistic network model with three types of paths. By defining the total cost minimal function, a linear planning model for the combined number was proposed. For solving this non-deterministic polynomial-hard problem, a mechanism of search based on neighborhood was investigated. Goovidnan et al^16^. formulated a multi-objective optimization model to develop a resilient and sustainable electricity SC network under uncertain conditions. The model simultaneously sought to minimize total costs, reduce factors that undermined resilience, and enhance selected dimensions of corporate social responsibility. To effectively manage uncertainty in electricity demand, they employed an innovative, robust framework that integrated robust optimization techniques with possibility theory based on fuzzy logic. Harafeh et al^17^. considered a CLSC network under uncertainty with the genetic algorithm, examined and solved it using a fuzzy planning model for reproduction. One of their goals was obtaining maximum profitability, and the other was calculation time. He^18^ enhanced the CLSC strategy by implementing a comprehensive optimisation approach that incorporated ecological, financial, and social effects via case-based probabilistic programming and the Lagrangian relaxation technique.

Jafarzadeh et al^19^. presented a multi-layer CLSC system that solved uncertainty in request and transport costs. Their methodology combined a mixed-integer linear programming (MILP) framework with robust-fuzzy optimization to enhance cost tactics, manufacturing queues, inventory control, and optimal mobility in SCs. Khorshidvand et al^20^. presented a CLSC model centered on the retrieval and remanufacturing of post-use products. Their model assessed various collecting tactics and determined ideal CLSC configurations suited to the requirements of original equipment manufacturers. Khot et al^21^. designed a mathematical model in reverse SC for optimizing production planning and control of inventory. In this model, the transportation and its cost parameter were not taken into account. This research aimed at reducing inventory and production cost, as well as increasing customer satisfaction and organizational profitability. Kuntz et al^22^. considered sustainability, resilience, robustness, and risk aversion approaches in designing a CLSC. They aimed to minimize costs, CO_2_ emissions, and energy consumption while maximizing career benefits.

### Fuzzy and robust approaches

Manikandan et al^23^. proposed a simulation-based optimization method, combined with reaction body methodology, to identify the appropriate support capabilities and the number of batches. The technique enhanced the approach’s flexibility regarding adaptability, robustness, and adaptability to market fluctuations. Mridha et al^24^. proposed a multi-objective approach to designing a viable blood supply chain that encompasses participants, collection and distribution centers, and clinics. The approach sought to ensure the reliability of the clinical blood supplies and promote social responsibility while minimizing costs and environmental impacts. To solve the model, they first applied an enhanced epsilon-constraint method to convert it into a single-objective form, followed by the use of an imperialist competitive algorithm, which was validated through a series of experimental case studies. Miyangaskary et al^25^. proposed a two-stage approach for designing a sustainable and resilient SC. Initially, they applied a fuzzy c-means clustering technique to assess the sustainability performance of potential suppliers. Subsequently, they developed a stochastic optimization technique designed to reduce the anticipated total cost while simultaneously maximizing aggregate sustainability performance under disruption scenarios.

Pak et al^26^. designed a multi-echelon, multi-objective, robust fuzzy CLSC design model that addresses uncertainty and incorporates all three dimensions of sustainability. This model simultaneously addresses total cost minimization, carbon constraints, and social impact maximization to achieve supply chain sustainability, effectively balancing multiple conflicting objectives.

Numerous studies have used NSGA-II and MOPSO to address multi-objective optimization problems within supply chain and sustainability frameworks. Rodríguez‐Escoto et al^27^. considered the NSGA-II for a sustainable closed-loop supply chain network, emphasizing cost and environmental objectives in deterministic conditions. Saeed et al^28^. utilized the NSGA-II to formulate a sustainable closed-loop supply chain with fixed demand and constrained uncertainty representation. Salçuk et al^29^. applied the MOPSO for multi-objective supply chain network design under uncertainty, although their model overlooked reverse logistics in battery systems and did not incorporate integrated social sustainability indicators.

Distinct from prior studies, this research addresses SC network design for companies handling high-value end-of-life products, explicitly accounting for potential disruptions in facilities, transportation routes, and customer satisfaction. The model incorporates decisions regarding which candidate nodes and links should be activated. Table 1 summarizes the existing research gaps by presenting a detailed comparison of related studies and offering a more precise classification of the subject area.

**Table 1:** Summary of research background on supply chain networks from various related works.

Authors	Type of network		Reliability considered		Uncertainty modeling approach				Type of periods		Objectives			Solution approaches		
	Open loop	Closed loop	Yes	No	Stochastic programming	Fuzzy	Simulation-based	Robust optimization	Single	Multi	Client satisfaction	Financial	Environmental	Exact	Heuristic	Metaheuristic
Sarkar et al^30^.		*						*		*	*		*	*		
Setiawan et al ^31^		*	*				*			*		*	*	*		
Soleimani et al^32^.	*		*			*	*		*		*					*
Tavana et al ^33^		*					*			*	*		*			*
Vali-Siar et al^34^.		*		*	*					*		*	*	*		
Vukojevic et al ^35^		*					*	*		*	*				*	
Zhang et al^36^.		*	*	*	*					*		*		*		
*This research*		*	*			*		*		*	*	*	*	*		*

### Research gaps

Based on the literature review and the insights presented, it is evident that many previous studies relied on overly simplistic models to formulate CLSC problems. Moreover, most of these models fail to adequately address potential sustainability risks, which can hinder their applicability in real-world scenarios. In recent years, sustainability concerns have garnered increasing global attention from both policymakers and the public, underscoring the urgent need to integrate environmental considerations into SCM. In this context, optimizing the recycling of returned products becomes a strategic priority. Additionally, since SC operations account for a substantial share of a product’s total cost, a thorough evaluation of SC processes can offer valuable opportunities for cost efficiency and performance improvement. Based on our understanding and the aforementioned assessment, there is only a limited number of studies focusing on strategic and operational selections in uncertain settings. Furthermore, client satisfaction, an important component of sustainability, has been largely overlooked in the literature. The implementation of CLSC network design has not been adequately explored within the battery sector.

## Problem description

In battery manufacturing companies, prioritizing environmental safety and resource efficiency is crucial due to its direct impact on economic sustainability and environmental quality. The following activities of the battery manufacturer are explicitly incorporated into the mathematical model of the proposed closed-loop supply chain design. Strategic choices about the site and capacity of production, collecting, recycling, and remanufacturing facilities are represented by binary and continuous decision variables. Material flows of batteries and reclaimed components in forward and reverse logistics are regulated by flow balance and capacity constraints. The economic, environmental, and social impacts associated with battery production and recovery processes are incorporated into the sustainability-focused objective functions, ensuring that the operational features of the battery closed-loop supply chain are accurately represented in the optimization framework.

**Fig. 1:**
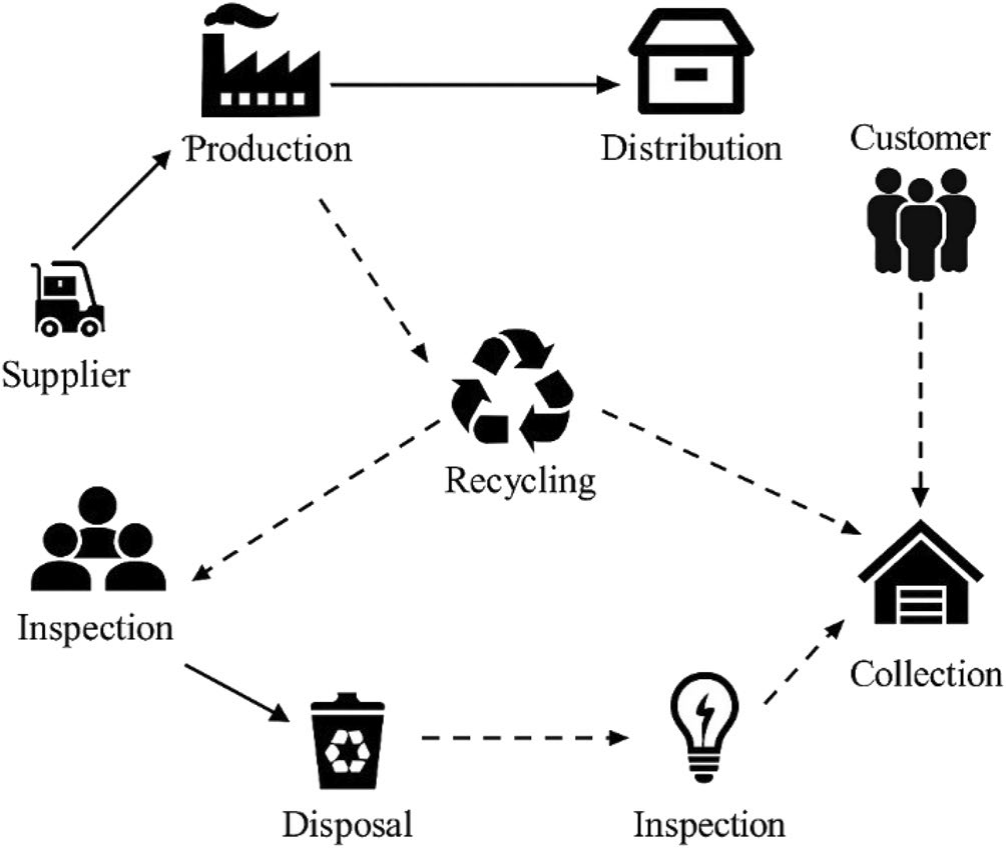
Proposed CLSC network.

Moreover, uncertainty in customer demand hinders evaluations of SC profitability, as it significantly impacts both operating expenses and revenue. The present research provides a multi-product, multi-period CLSC framework specifically designed for the battery sector. The network structure comprises suppliers, manufacturing facilities, distribution centers, and clients in the forward movement. It incorporates collecting, inspection, recycling, and disposal centers into the reverse flow (refer to Fig. 1). Raw ingredients are sourced from vendors and subsequently transformed into finished batteries at manufacturing facilities. These products are then transferred to distributors, where the majority are shipped expeditiously due to their short shelf life. Nonetheless, during transit, certain batteries may sustain damage due to insufficient packaging, improper handling, or equipment malfunction. Batteries in distribution centers are either dispatched to consumers if they meet quality standards or returned to collection facilities if they are damaged or expired. In the reverse flow, returned batteries from clients and unwanted or discontinued goods from distributors are directed to collection centers for evaluation, recycling, or ultimate disposal.

### Assumptions

The additional essential assumptions included in our proposed approach are as follows:

- Supplier warehousing costs are excluded from the model (Eqs. (2), (7)).
- Each production center manages inventory independently, with no inter-center transfers (Eqs. (16)–(17)).
- Transportation costs are assumed to be time-invariant across planning periods (Eq. (1)).
- Transportation operations may use either purchased or leased vehicles, subject to capacity constraints (Eqs. (23)–(24), (25)–(26), (27)–(28)).
- Customer demand, fulfillment, and shortages are explicitly modeled by product category (Eqs. (8), (29)–(30)).
- Inventory shortages are permitted and incorporated through balance constraints and penalty terms (Eqs. (29)–(30), Eq. (3)).
- Disposal/burial and recycling facilities are considered as strategic location decisions in the closed-loop network (Eqs. (35)–(36)).

### Model formulation

Notations are provided in Appendix-A. The first objective function is obtained by the goal of minimization of total costs of transportation according to Eq. (1). Maintenance, disposal and burial cost is achieved by Eq. (2). Shortage cost is obtained by Eq. (3). The localization cost is calculated by Eq. (4). Construction of the disposal and burial and recycling centers is calculated by Eq. (5). Purchase and production cost is minimized in Eq. (6).

1 $$\begin{aligned} TTC & = \sum {_{{(K = 1)}}^{K} } \sum {_{{(S = 1)}}^{S} } \sum {_{{(M = 1)}}^{M} } \sum {_{{(V = 1)}}^{V} } \sum {_{{(T = 1)}}^{T} } C1_{{ksmv}} q1_{{ksmtv}} \\ & + \sum {_{{(M = 1)}}^{M} } \sum {_{{(D = 1)}}^{D} } \sum {_{{(V = 1)}}^{V} } \sum {_{{(T = 1)}}^{T} } \sum {_{{(P = 1)}}^{P} } C2_{{pmdv}} q2_{{pmdtv}} \\ & + \sum {_{{(P = 1)}}^{P} } \sum {_{{(D = 1)}}^{D} } \sum {_{{(C = 1)}}^{C} } \sum {_{{(V = 1)}}^{V} } \sum {_{{(T = 1)}}^{T} } C3_{{pdcv}} q3_{{pdctv}} \\ & + \sum {_{{(P = 1)}}^{P} } \sum {_{{(D = 1)}}^{D} } \sum {_{{(R = 1)}}^{R} } \sum {_{{(V = 1)}}^{V} } \sum {_{{(T = 1)}}^{T} } C4_{{pdrv}} q4_{{pdrtv}} \\ & + \sum {_{{(P = 1)}}^{P} } \sum {_{{(D = 1)}}^{D} } \sum {_{{(I = 1)}}^{I} } \sum {_{{(V = 1)}}^{V} } \sum {_{{(T = 1)}}^{T} } C5_{{pdlv}} q5_{{pdltv}} \\ & + \sum {_{{(K = 1)}}^{K} } \sum {_{{(S = 1)}}^{S} } \sum {_{{(I = 1)}}^{I} } \sum {_{{(V = 1)}}^{V} } \sum {_{{(T = 1)}}^{T} } C6_{{kIsv}} q6_{{kIstv}} \\ & + \sum {_{{(K = 1)}}^{K} } \sum {_{{(S = 1)}}^{S} } \sum {_{{(M = 1)}}^{M} } \sum {_{{(W = 1)}}^{W} } \sum {_{{(T = 1)}}^{T} } CC1_{{ksmw}} qq1_{{ksmtv}} \\ & + \sum {_{{(M = 1)}}^{M} } \sum {_{{(D = 1)}}^{D} } \sum {_{{(W = 1)}}^{W} } \sum {_{{(T = 1)}}^{T} } \sum {_{{(P = 1)}}^{P} } CC2_{{pmdw}} qq2_{{pmdtw}} \\ & + \sum {_{{(P = 1)}}^{P} } \sum {_{{(D = 1)}}^{D} } \sum {_{{(C = 1)}}^{C} } \sum {_{{(W = 1)}}^{W} } \sum {_{{(T = 1)}}^{T} } CC3_{{pdcw}} qq3_{{pdctw}} \\ & + \sum {_{{(P = 1)}}^{P} } \sum {_{{(D = 1)}}^{D} } \sum {_{{(R = 1)}}^{R} } \sum {_{{(V = 1)}}^{V} } \sum {_{{(T = 1)}}^{T} } CC4_{{pdrv}} qq4_{{pdrtv}} \\ & + \sum {_{{(P = 1)}}^{P} } \sum {_{{(D = 1)}}^{D} } \sum {_{{(I = 1)}}^{I} } \sum {_{{(V = 1)}}^{V} } \sum {_{{(T = 1)}}^{T} } CC5_{{pdIv}} qq5_{{pdItv}} \\ & + \sum {_{{(K = 1)}}^{K} } \sum {_{{(S = 1)}}^{S} } \sum {_{{(I = 1)}}^{I} } \sum {_{{(V = 1)}}^{V} } \sum {_{{(T = 1)}}^{T} } CC6_{{kIsv}} qq6_{{kIstv}} \\ \end{aligned}$$

2 $$\begin{aligned} THC & = \sum\nolimits_{{k = 1}}^{K} {\sum\nolimits_{{m = 1}}^{M} {\sum\nolimits_{{t = 1}}^{T} {h1_{{km}} i1_{{kmt}} } } } \\ & + \sum\nolimits_{{m = 1}}^{M} {\sum\nolimits_{{t = 1}}^{T} {\sum\nolimits_{{p = 1}}^{P} {h2_{{pm}} i2_{{pmt}} } } } \\ & + \sum\nolimits_{{d = 1}}^{D} {\sum\nolimits_{{t = 1}}^{T} {\sum\nolimits_{{p = 1}}^{P} {h3_{{pd}} } } } i3_{{pdt}} \\ & + \sum\nolimits_{{p = 1}}^{P} {\sum\nolimits_{{d = 1}}^{D} {\sum\nolimits_{{r = 1}}^{R} {\sum\nolimits_{{t = 1}}^{T} {\sum\nolimits_{{w = 1}}^{W} {h4_{{pr}} } } } } } qq4_{{pdrtw}} \\ & + \sum\nolimits_{{p = 1}}^{P} {\sum\nolimits_{{d = 1}}^{D} {\sum\nolimits_{{l = 1}}^{L} {\sum\nolimits_{{t = 1}}^{T} {\sum\nolimits_{{w = 1}}^{W} {h5_{{pl}} qq5_{{pdltw}} } } } } } \\ \end{aligned}$$

3 $$\begin{aligned} TSC & = \sum\nolimits_{{k = 1}}^{K} {\sum\nolimits_{{m = 1}}^{M} {\sum\nolimits_{{t = 1}}^{T} {\pi 3_{{km}} b1_{{kmt}} } } } \\ & + \sum\nolimits_{{d = 1}}^{D} {\sum\nolimits_{{t = 1}}^{T} {\sum\nolimits_{{p = 1}}^{P} {\pi 1_{{pd}} b2_{{pdt}} } } } \\ & + \sum\nolimits_{{c = 1}}^{C} {\sum\nolimits_{{t = 1}}^{T} {\sum\nolimits_{{p = 1}}^{P} {\pi 2_{{pc}} b3_{{pct}} } } } \\ \end{aligned}$$

4 $$TOC = \sum\nolimits_{{m = 1}}^{M} {\sum\nolimits_{{i = 1}}^{I} {f1_{{mi}} x1_{{mi}} } } + \sum\nolimits_{{d = 1}}^{D} {\sum\nolimits_{{j = 1}}^{J} {f2_{{dj}} x2_{{dj}} } }$$

5 $$TRC = \sum\nolimits_{{r = 1}}^{R} {f3_{r} x3_{r} } + \sum\nolimits_{{l = 1}}^{L} {f4_{r} x10_{i} }$$

6 $$\begin{aligned} TPC & = \sum\nolimits_{{k = 1}}^{K} {\sum\nolimits_{{s = 1}}^{S} {\sum\nolimits_{{m = 1}}^{M} {\sum\nolimits_{{t = 1}}^{T} {\sum\nolimits_{{v = 1}}^{V} {p1_{{ksm}} q1_{{ksmtv}} } } } } } \\ & + \sum\nolimits_{{m = 1}}^{M} {\sum\nolimits_{{d = 1}}^{D} {\sum\nolimits_{{t = 1}}^{T} {\sum\nolimits_{{v = 1}}^{V} {\sum\nolimits_{{p = 1}}^{P} {p2_{{pmd}} q2_{{pmdtv}} } } } } } \\ & - \sum\nolimits_{{p = 1}}^{P} {\sum\nolimits_{{d = 1}}^{D} {\sum\nolimits_{{c = 1}}^{C} {\sum\nolimits_{{t = 1}}^{T} {\sum\nolimits_{{v = 1}}^{V} {p3_{{pdc}} q3_{{pdctv}} } } } } } \\ & + \sum\nolimits_{{k = 1}}^{K} {\sum\nolimits_{{s = 1}}^{S} {\sum\nolimits_{{m = 1}}^{M} {\sum\nolimits_{{t = 1}}^{T} {\sum\nolimits_{{v = 1}}^{V} {p1_{{ksm}} qq1_{{ksmtw}} } } } } } \\ & + \sum\nolimits_{{m = 1}}^{M} {\sum\nolimits_{{d = 1}}^{D} {\sum\nolimits_{{t = 1}}^{T} {\sum\nolimits_{{w = 1}}^{W} {\sum\nolimits_{{p = 1}}^{P} {p2_{{pmd}} qq2_{{pmdtw}} } } } } } \\ & - \sum\nolimits_{{p = 1}}^{P} {\sum\nolimits_{{d = 1}}^{D} {\sum\nolimits_{{c = 1}}^{C} {\sum\nolimits_{{t = 1}}^{T} {\sum\nolimits_{{w = 1}}^{W} {p3_{{pdc}} qq3_{{pdctw}} } } } } } \\ & + \sum\nolimits_{{m = 1}}^{M} {\sum\nolimits_{{t = 1}}^{T} {e_{{pm}} q4_{{pmt}} } } \\ \end{aligned}$$

7 $$\mathit{Min} zz1=TTC+THC+TSC+TOC+TRC+TPC$$

The main first objective of the function Eq. (7) reduces the overall economic cost of the closed-loop supply chain network. This objective consolidates transportation costs (TTC), inventory holding costs (THC), shortage costs (TSC), facility opening costs (TOC), recovery and disposal costs (TRC), and production-related costs (TPC), thus expressing the economic aspect of sustainability.

The second objective function Eq. (8) maximizes customer satisfaction by reducing unmet demand across distribution centers.

8 $$Max zz2=\sum_{m=1}^{M}\sum_{t=1}^{T}\sum_{p=1}^{P} \frac{{i1}_{pdt}}{{\widetilde{TD}}_{pct}}$$

The third objective function Eqs. (9–15) minimizes environmental impacts associated with transportation activities, considering emissions generated by both purchased and leased vehicles.

9 $$\begin{aligned} PP_{1} & = \sum\nolimits_{{k = 1}}^{K} {\sum\nolimits_{{s = 1}}^{S} {\sum\nolimits_{{m = 1}}^{M} {\sum\nolimits_{{t = 1}}^{T} {\sum\nolimits_{{v = 1}}^{V} {\left[ {\frac{{q1_{{ksmtv}} }}{{v1_{v} }}} \right]} } } } } \left( {\left. {\alpha 2_{v} - \alpha 1_{v} } \right)} \right.l1_{{sm}} \\ & + \sum\nolimits_{{k = 1}}^{K} {\sum\nolimits_{{s = 1}}^{S} {\sum\nolimits_{{m = 1}}^{M} {\sum\nolimits_{{t = 1}}^{T} {\sum\nolimits_{{w = 1}}^{W} {\left[ {\frac{{qq1_{{ksmtv}} }}{{vv1_{v} }}} \right]} } } } } \left( {\left. {\alpha \alpha 2_{w} - \alpha \alpha 1_{w} } \right)} \right.l1_{{sm}} \\ \end{aligned}$$

10 $$\begin{aligned} PP_{2} & = \sum\nolimits_{{d = 1}}^{D} {\sum\nolimits_{{m = 1}}^{M} {\sum\nolimits_{{t = 1}}^{T} {\sum\nolimits_{{v = 1}}^{V} {\sum\nolimits_{{p = 1}}^{P} {\left[ {\frac{{q2_{{pmdtv}} }}{{v1_{v} }}} \right]} } } } } \left( {\left. {\alpha 2_{v} - \alpha 1_{v} } \right)} \right.l2_{{md}} \\ & + \sum\nolimits_{{d = 1}}^{D} {\sum\nolimits_{{m = 1}}^{M} {\sum\nolimits_{{t = 1}}^{T} {\sum\nolimits_{{w = 1}}^{W} {\sum\nolimits_{{p = 1}}^{P} {\left[ {\frac{{qq2_{{pmdtw}} }}{{vv1_{v} }}} \right]} } } } } \left( {\left. {\alpha \alpha 2_{w} - \alpha \alpha 1_{w} } \right)} \right.l2_{{md}} \\ \end{aligned}$$

11 $$\begin{aligned} PP_{3} & = \sum\limits_{{d = 1}}^{D} {\sum\nolimits_{{m = 1}}^{M} {\sum\nolimits_{{t = 1}}^{T} {\sum\nolimits_{{v = 1}}^{V} {\sum\nolimits_{{p = 1}}^{P} {\left[ {\frac{{q3_{{pdctv}} }}{{v1_{v} }}} \right]} } } } } \left( {\left. {\alpha 2_{v} - \alpha 1_{v} } \right)} \right.l3_{{dc}} \\ & + \sum\nolimits_{{d = 1}}^{D} {\sum\nolimits_{{m = 1}}^{M} {\sum\nolimits_{{t = 1}}^{T} {\sum\nolimits_{{w = 1}}^{W} {\sum\nolimits_{{p = 1}}^{P} {\left[ {\frac{{qq3_{{pdctw}} }}{{vv1_{w} }}} \right]} } } } } \left( {\left. {\alpha \alpha 2_{w} - \alpha \alpha 1_{w} } \right)} \right.l3_{{dc}} \\ \end{aligned}$$

12 $$\begin{aligned} PP_{4} & = \sum\nolimits_{{d = 1}}^{D} {\sum\nolimits_{{r = 1}}^{R} {\sum\nolimits_{{t = 1}}^{T} {\sum\nolimits_{{v = 1}}^{V} {\sum\nolimits_{{p = 1}}^{P} {\left[ {\frac{{q4_{{pdrtw}} }}{{v1_{v} }}} \right]} } } } } \left( {\left. {\alpha 2_{v} - \alpha 1_{v} } \right)} \right.l4_{{dr}} \\ & + \sum\nolimits_{{d = 1}}^{D} {\sum\nolimits_{{r = 1}}^{R} {\sum\nolimits_{{t = 1}}^{T} {\sum\nolimits_{{w = 1}}^{W} {\sum\nolimits_{{p = 1}}^{P} {\left[ {\frac{{qqr_{{pdrtw}} }}{{vv1_{w} }}} \right]} } } } } \left( {\left. {\alpha \alpha 2_{w} - \alpha \alpha 1_{w} } \right)} \right.l4_{{dr}} \\ \end{aligned}$$

13 $$\begin{aligned} PP_{5} = & \sum\nolimits_{{d = 1}}^{D} {\sum\nolimits_{{l = 1}}^{L} {\sum\nolimits_{{t = 1}}^{T} {\sum\nolimits_{{v = 1}}^{V} {\sum\nolimits_{{p = 1}}^{P} {\left[ {\frac{{q5_{{pdltv}} }}{{v1_{v} }}} \right]} } } } } \left( {\left. {\alpha 2_{v} - \alpha 1_{v} } \right)} \right.l5_{{dl}} \\ & + \sum\nolimits_{{d = 1}}^{D} {\sum\nolimits_{{l = 1}}^{L} {\sum\nolimits_{{t = 1}}^{T} {\sum\nolimits_{{w = 1}}^{W} {\sum\nolimits_{{p = 1}}^{P} {\left[ {\frac{{qq5_{{pdltw}} }}{{vv1_{w} }}} \right]} } } } } \left( {\left. {\alpha \alpha 2_{w} - \alpha \alpha 1_{w} } \right)} \right.l5_{{dl}} \\ \end{aligned}$$

14 $$\begin{aligned} PP_{6} & = \sum\nolimits_{{L = 1}}^{L} {\sum\nolimits_{{s = 1}}^{S} {\sum\nolimits_{{t = 1}}^{T} {\sum\nolimits_{{v = 1}}^{V} {\sum\nolimits_{{k = 1}}^{K} {\left[ {\frac{{q6_{{ksltv}} }}{{v1_{v} }}} \right]} } } } } \left( {\left. {\alpha 2_{v} - \alpha 1_{v} } \right)} \right.l6_{{ls}} \\ & + \sum\nolimits_{{l = 1}}^{L} {\sum\nolimits_{{s = 1}}^{S} {\sum\nolimits_{{t = 1}}^{T} {\sum\nolimits_{{w = 1}}^{W} {\sum\nolimits_{{k = 1}}^{K} {\left[ {\frac{{qq6_{{ksltw}} }}{{vv1_{w} }}} \right]} } } } } \left( {\left. {\alpha \alpha 2_{w} - \alpha \alpha 1_{w} } \right)} \right.l6_{{dlls}} \\ \end{aligned}$$

15 $$\mathit{Min}zz3={PP}_{1}+{PP}_{2}+{PP}_{3}+{PP}_{4}+{PP}_{5}+{PP}_{6}$$

This study addresses sustainability by concurrently evaluating economic, social, and environmental objectives within a cohesive multi-objective optimization framework. The economic purpose aims to minimize the entire system cost, encompassing transportation, holding, shortage, operational, recovery, and production expenses (Eqs. (1)–(2)). The social objective aims to augment consumer pleasure by minimizing unmet demand and elevating service levels throughout distribution centers (Eq. (8)). The environmental purpose seeks to reduce the ecological effects linked to transportation and logistics activities, encompassing emissions from both owned and leased vehicles (Eqs. (9)–(10)).

These objectives are intrinsically contradictory: minimizing costs may diminish service quality or escalate environmental repercussions, whereas enhanced customer satisfaction or reduced emissions generally necessitate increased resources and investment. The suggested model integrates the three sustainability objectives into a multi-objective decision-making framework, enabling decision-makers to identify Pareto-efficient solutions that reflect varying sustainability priorities.

The constraints comprised in the proposed CLSC network design for the battery industry.

are expressed as follows. In Eq. (16), the constraint of inventory balance of final product in any center is shown.

16 $$i2_{{pmt}} = i2_{{pm(t - 1)}} + q4_{{pmt}} - \sum\nolimits_{{d = 1}}^{D} {\sum\nolimits_{{v = 1}}^{V} {q2_{{pmdtv}} } } - \sum\nolimits_{{d = 1}}^{D} {\sum\nolimits_{{w = 1}}^{W} {qq2_{{pmdtw}} } } ,\forall p,m,t$$

17 $$i1_{{kmt}} = i1_{{km(t - 1)}} + \sum\nolimits_{{s = 1}}^{S} {\sum\nolimits_{{v = 1}}^{V} {q1_{{ksmtv}} } } + \sum\nolimits_{{s = 1}}^{S} {\sum\nolimits_{{w = 1}}^{W} {qq1_{{ksmtw}} } } ,\forall k,m,t$$

18 $$i3_{{pdt}} = i3_{{pd(t - 1)}} + \sum\nolimits_{{m = 1}}^{M} {\sum\nolimits_{{v = 1}}^{V} {\sum\nolimits_{{p = 1}}^{P} {q2_{{pmdtv}} } } } - \sum\nolimits_{{c = 1}}^{C} {\sum\nolimits_{{v = 1}}^{V} {q3_{{pdctv}} } } + ,\forall p,d,t$$

Constraints (16)–(18) define the inventory balance relationships for raw materials and finished products at production and distribution centers. These equations ensure flow conservation by linking inventory levels between consecutive periods with incoming shipments, outgoing flows, and shortage quantities.

Constraints (19)–(22) impose production and storage capacity limits at each facility, ensuring that material flows and inventory levels do not exceed available operational capacities.

19 $$\sum\nolimits_{{m = 1}}^{M} {\sum\nolimits_{{p = 1}}^{P} {PT_{{pm}} q4_{{pmt}} } } + \sum\nolimits_{{p = 1}}^{P} {\sum\nolimits_{{m = 1}}^{M} {ST_{{pm}} X0_{{pmt}} } } \le TT_{t} ,\forall t$$

20 $$\sum\nolimits_{{p = 1}}^{P} {\left( {\left. {i2_{{pm(t - 1)}} + q4_{{pmt}} } \right)g2_{{pmt}} \le v3_{m} X1_{{mi}} ,\forall m,i,t} \right.}$$

21 $$\sum\nolimits_{{k = 1}}^{K} {\left( {\left. {\sum\nolimits_{{s = 1}}^{S} {\sum\nolimits_{{v = 1}}^{V} {q1_{{ksmtv}} } } + \sum\nolimits_{{s = 1}}^{S} {\sum\nolimits_{{w = 1}}^{W} {qq1_{{ksmtw}} + i1_{{km(t - 1)}} } } } \right)} \right.} g1_{k} \le v2_{m} X1_{{mi}} ,\forall m,i,t$$

22 $$\sum\nolimits_{{p = 1}}^{P} {\left( {\left. {\sum\nolimits_{{m = 1}}^{M} {\sum\nolimits_{{v = 1}}^{V} {q2_{{pmdtv}} } } \sum\limits_{{m = 1}}^{M} {\sum\limits_{{v = 1}}^{V} {} } + \sum\nolimits_{{m = 1}}^{M} {\sum\nolimits_{{w = 1}}^{W} {qq2_{{pmdtw}} } } + i3_{{pd(t - 1)}} } \right)} \right.} g2_{p} \le v4_{d} X2_{{dj}} ,\forall d,t,j$$

Constraints (23)–(28) restrict shipment quantities based on the capacity of purchased transportation vehicles.

23 $$\sum\nolimits_{{k = 1}}^{K} {\sum\nolimits_{{m = 1}}^{M} {q1_{{ksmtv}} g1_{k} \le v1_{v} ,\forall t,v,s} }$$

24 $$\sum\nolimits_{{p = 1}}^{P} {\sum\nolimits_{{d = 1}}^{D} {q2_{{pmdtv}} g2_{p} \le v1_{v} ,\forall t,m,v} }$$

25 $$\sum\nolimits_{{p = 1}}^{P} {\sum\nolimits_{{c = 1}}^{C} {q3_{{pdctv}} g2_{p} \le v1_{v} ,\forall t,d,v} }$$

26 $$\sum\nolimits_{{p = 1}}^{P} {\sum\nolimits_{{r = 1}}^{R} {q4_{{pdrtv}} g2_{p} \le v1_{v} ,\forall t,d,v} }$$

27 $$\sum\nolimits_{{p = 1}}^{P} {\sum\nolimits_{{l = 1}}^{L} {q5_{{pdltv}} g2_{p} \le v1_{v} ,\forall t,d,v} }$$

28 $$\sum\nolimits_{{k = 1}}^{K} {\sum\nolimits_{{l = 1}}^{L} {q6_{{klstv}} g1_{k} \le v1_{v} ,\forall t,s,v} }$$

Constraints (29)–(30) model customer demand satisfaction and allowable shortages at distribution centers. These equations permit unmet demand while accounting for it explicitly through shortage variables and associated penalty costs.

29 $$b3_{{pct}} = b3_{{pc(t - 1)}} + {\widetilde{TD}}_{{pct}} - \sum\nolimits_{{v = 1}}^{V} {\sum\nolimits_{{d = 1}}^{D} {q3_{{pdctv}} } } - \sum\nolimits_{{w = 1}}^{W} {\sum\nolimits_{{d = 1}}^{D} {qq3_{{pdctw}} } } ,\forall p,c,t$$

30 $$\begin{aligned} b2_{{pct}} & = b2_{{pc(t - 1)}} + \sum\nolimits_{{v = 1}}^{V} {\sum\nolimits_{{c = 1}}^{C} {q3_{{pdctv}} } } \\ & - \sum\nolimits_{{V = 1}}^{V} {\sum\nolimits_{{M = 1}}^{M} {\sum\nolimits_{{p = 1}}^{P} {q2_{{pmdtv}} } } } \\ & + \sum\nolimits_{{w = 1}}^{W} {\sum\nolimits_{{c = 1}}^{C} {qq3_{{pdctw}} } } \\ & - \sum\nolimits_{{V = 1}}^{V} {\sum\nolimits_{{M = 1}}^{M} {\sum\nolimits_{{p = 1}}^{P} {qq2_{{pmdtv}} } } } ,\forall p,t,d \\ \end{aligned}$$

Constraints (31)–(34) define the strategic facility location decisions in the CLSC network. Constraints (31) and (33) specify the required number of distribution centers and production centers to be established, respectively. Constraints (32) and (34) ensure that each candidate site can host at most one facility, preventing multiple assignments to the same location.

31 $$\sum\nolimits_{{D = 1}}^{D} {\sum\nolimits_{{j = 1}}^{J} {X2_{{dj}} = R1} }$$

32 $$\sum\nolimits_{{j = 1}}^{J} {X2_{{dj}} \le 1,\forall d}$$

33 $$\sum\nolimits_{{i = 1}}^{I} {\sum\nolimits_{{m = 1}}^{M} {X1_{{mi}} = R3} }$$

34 $$\sum\nolimits_{{m = 1}}^{M} {X1_{{mi}} \le 1,\forall i}$$

Constraints (35)–(36) define the binary location decisions for recycling and disposal facilities, determining whether these facilities are opened as part of the CLSC network design.

35 $$\sum\nolimits_{{r - 1}}^{R} {X3_{r} = R2}$$

36 $$\sum\nolimits_{{l = 1}}^{L} {X10_{l} = R5}$$

Constraints (37)–(42) confirm the viability of vehicle allocation for each transportation arc. They specifically restrict the assignment and activation variables related to vehicle purchases along the supplier-to-producer, distributor-to-customer, producer-to-distributor, distributor-to-disposal, distributor-to-recycling, and disposal-to-supplier routes, respectively.

37 $$\sum\nolimits_{{v = 1}}^{V} {X4_{{ksmtv}} \le 1,\forall k,s,m,s}$$

38 $$\sum\nolimits_{{v = 1}}^{V} {X6_{{pdctv}} \le 1,\forall p,d,c,t}$$

39 $$\sum\nolimits_{{v = 1}}^{V} {X5_{{pmdtv}} \le 1,\forall p,d,m,t}$$

40 $$\sum\nolimits_{{v = 1}}^{V} {X7_{{pdrtv}} \le 1,\forall p,d,r,t}$$

41 $$\sum\nolimits_{{v = 1}}^{V} {X8_{{pdltv}} \le 1,\forall p,d,l,t}$$

42 $$\sum\nolimits_{{v = 1}}^{V} {X9_{{klstv}} \le 1,\forall k,l,s,t}$$

Constraints (43)-(45) connects production output with outward shipments. It guarantees that the cumulative amount dispatched from a manufacturing facility (summed across distributors and vehicle categories). Also, it indicates the material balance and availability condition for raw resources.

43 $$\sum\nolimits_{{v = 1}}^{V} {\sum\nolimits_{{d = 1}}^{D} {q2_{{pmdtv}} \le q4_{{pmt}} ,\forall p,m,t} }$$

44 $$b1_{{kmt}} = b1_{{km(t - 1)}} - \sum\nolimits_{{v = 1}}^{V} {\sum\nolimits_{{s = 1}}^{S} {q1_{{ksmtv}} } } ,\forall m,k,t$$

45 $$\sum\nolimits_{{k = 1}}^{K} {\sum\nolimits_{{m = 1}}^{M} {qq1_{{ksmtw}} } } \le v1_{w} ,\forall t,w,s$$

Constraints (46)–(50) impose capacity constraints on rental vehicles across several transportation connections within the closed-loop supply chain, encompassing producer–distributor, distributor–customer, distributor–disposal, distributor–recycling, and recycling–supplier routes. These limits ensure that the quantities of transported products do not exceed the rental vehicle’s available capacity during each session. Constraint (51) limits the allocation of rental automobiles to the supplier–producer route to a maximum of one vehicle per route and time period.

46 $$\sum\nolimits_{{p = 1}}^{P} {\sum\nolimits_{{d = 1}}^{D} {qq2_{{pdmtw}} \le vv1_{w} ,\forall t,d,w} }$$

47 $$\sum\nolimits_{{p = 1}}^{P} {\sum\nolimits_{{c = 1}}^{C} {qq3_{{pctdw}} \le vv1_{w} ,\forall t,d,w} }$$

48 $$\sum\nolimits_{{p = 1}}^{P} {\sum\nolimits_{{r = 1}}^{R} {qq4_{{pdrtw}} } } \le vv1_{w} ,\forall t,d,w$$

49 $$\sum\nolimits_{{p = 1}}^{P} {\sum\nolimits_{{l = 1}}^{L} {qq5_{{pdltw}} } } \le vv1_{w} ,\forall t,d,w$$

50 $$\sum\nolimits_{{k = 1}}^{K} {\sum\nolimits_{{l = 1}}^{L} {qq6_{{klstw}} \le vv1_{w} ,\forall t,s,w} }$$

51 $$\sum\nolimits_{{w = 1}}^{W} {xx4_{{ksmtw}} } \le 1,\forall k,s,m,t$$

Constraints (52)–(55) limit the number of rented vehicles assigned to different transportation routes in the CLSC network. Specifically, these constraints ensure that at most one rented vehicle can be allocated to each distributor–customer, producer–distributor, distributor–disposal, and distributor–recycling route in each period. This formulation avoids multiple vehicle assignments on a single route and maintains operational feasibility of rented vehicle deployment.

52 $$\sum\nolimits_{{w = 1}}^{W} {xx6_{{pdctw}} \le 1,\forall p,d,c,t}$$

53 $$\sum\nolimits_{{w = 1}}^{W} {xx5_{{pmdtw}} \le 1,\forall p,d,m,t}$$

54 $$\sum\nolimits_{{w = 1}}^{W} {xx7_{{pdrtw}} \le 1,\forall p,d,r,t}$$

55 $$\sum\nolimits_{{w = 1}}^{W} {xx8_{{pdltw}} } \le 1,\forall p,d,l,t$$

Constraints (56)–(61) limit the quantities of forward and reverse shipments handled by procured vehicles, ensuring that the conveyed flow on each route remains within the designated vehicle capacity, which is determined by the product of the number of vehicles and the capacity variable $LN$. Constraints (62)–(67) impose identical capacity-feasibility requirements for leased vehicles, ensuring that shipment volumes remain within permissible limits across all routes. These limitations collectively connect demand-driven flows to fleet allocation decisions and ensure transportation feasibility across the CLSC network.

56 $${q1}_{ksmtv}\le {LN.x4}_{ksmtv}, \forall k,s,m,v,t$$

57 $${q3}_{pdctv}\le LN.{x6}_{pdctv}, \forall p,d,c,t,v$$

58 $$q2_{pmdtv} \le LN.x5_{pmdtv} , \forall p,d,m,t,v$$

59 $$q4_{pdrtv} \le LN.x7_{pdrtv} , \forall p,d,r,t,v$$

60 $$q5_{pdltv} \le LN.x8_{pdItv} , \forall p,d,l,t,v$$

61 $$q6_{klstv} \le LN.x9_{klstv} , \forall k,s,l,t,v$$

62 $$qq1_{ksmtw} \le LN.xx4_{ksmtw} , \forall k,s,m,w,t$$

63 $$qq3_{pdctw} \le LN.xx6_{pdctw} , \forall p,d,c,t,w$$

64 $$qq2_{pmdtw} \le LN.xx5_{pmdtw} , \forall p,m,d,t,w$$

65 $$qq4_{pdrtw} \le LN.xx7_{pdrtw} , \forall p,d,r,t,w$$

66 $$qq5_{pdltw} \le LN.xx8_{pdltw} , \forall p,d,l,t,w$$

67 $$qq6_{klstw} \le LN.xx9_{klstw} , \forall k,s,l,t,w$$

In Eq. (68), the vehicles from supplier to producer can move from the mentioned producer to other producers.

68 $$q1_{ksmtv} \le LN\left( {xx7} \right._{ksmtv} + x4_{km\mathop m\limits tv} ), \forall k,s,m,\mathop m\limits ,t,v$$

Equations (69) − (70) depict domains of other decision variables.

69 $$\begin{aligned} & q1_{{ksmtv}} ,q2_{{pmdtv}} ,q3_{{pdctv}} ,q4_{{pdrtv}} ,q5_{{pdltv}} ,q6_{{klstv}} ,qq1_{{ksmtw}} ,qq2_{{pmdtw}} , \\ & qq3_{{pdctw}} ,qq4_{{pdrtw}} ,qq5_{{pdltw}} ,qq6_{{klstw}} \ge 0,\forall k,s,m,t,p,r,d,l,w,c,v \\ \end{aligned}$$

70 $$X1_{mi} . X2_{mi} . X3_{mi} . X4_{mi} . X5_{mi} . X6_{mi} . X7_{mi} . X8_{mi} \in \left\{ {0,1} \right\}, \forall m,i$$

## Solution approaches

**Fig. 2:**
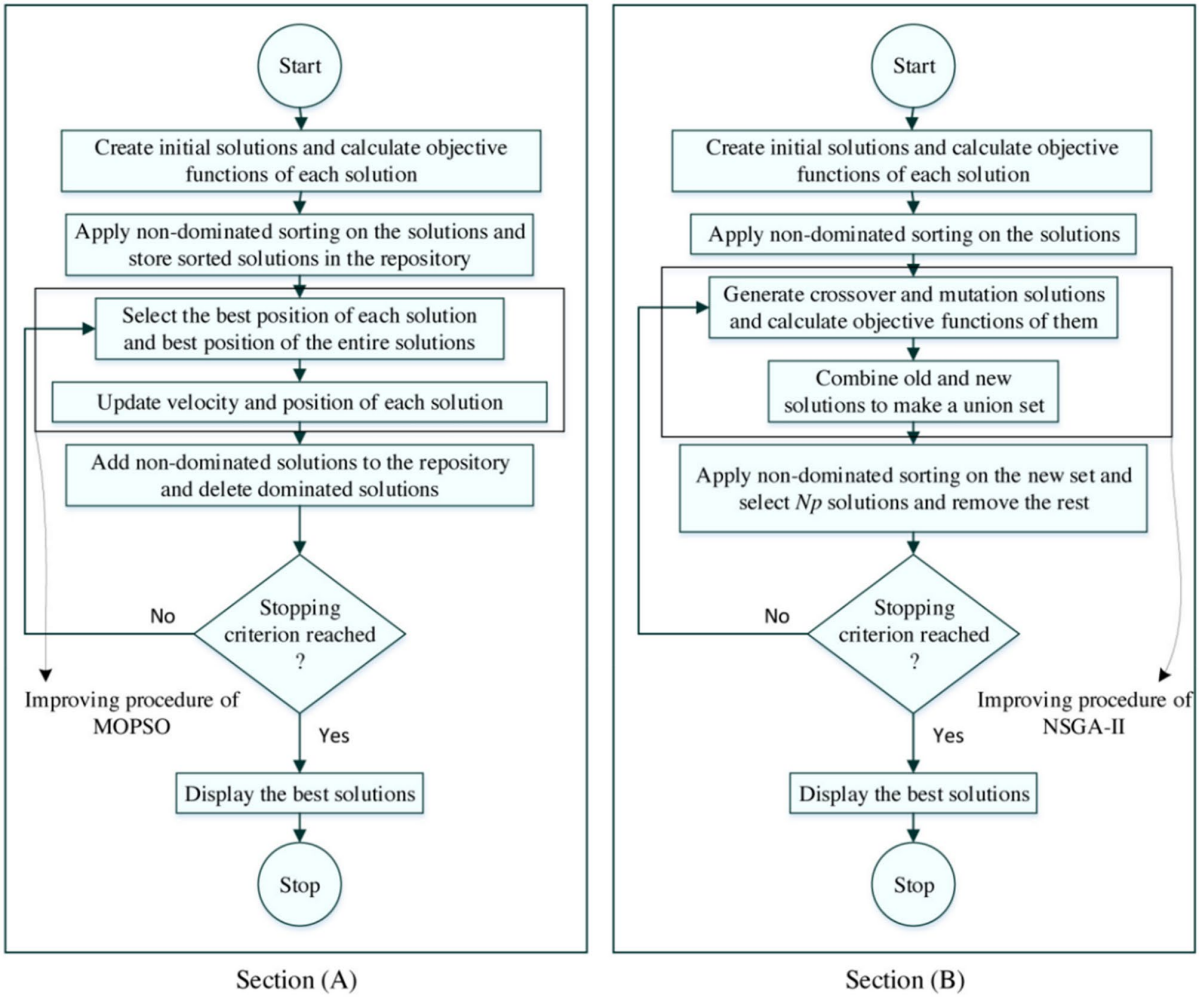
Flowchart of proposed two-stage metaheuristic.

This section employs the sophisticated metaheuristic solution methodologies (i.e., NSGA-II and MOPSO) to address a sharp alternative to the fuzzy multi-objective CLSC framework. These algorithms are chosen for their shown efficacy in addressing large-scale, nonlinear, and unpredictable optimization challenges. Their performance is assessed based on essential objective function parameters, including overall cost, customer satisfaction, and environmental impact. The comparison of results identifies the most successful algorithm, which is employed as the central solution aspect in our presented methodology in Fig. 2.

Figure 2 illustrates the workflow of the enhanced MOPSO and NSGA-II algorithms used to address the proposed CLSC network design issue. As seen in Section (A), the enhanced MOPSO algorithm begins by generating an initial population of solutions and evaluating their goal functions. Subsequently, non-dominated sorting is executed, and the optimal individual and global locations are recognized and recorded in an external repository. Particle velocities and locations are updated iteratively, retaining only non-dominated solutions in the repository to guide the search. This technique is reiterated until the termination requirement is fulfilled. Section (B) describes the workflow of the NSGA-II algorithm, wherein novel offspring solutions are produced using crossover and mutation operators. The parent and progeny populations are combined, followed by non-dominated sorting and selection to form the subsequent generation. This iterative procedure persists until the termination criterion is satisfied.

The novelty of the suggested enhancement is in the utilization of an external non-dominated solution repository, coupled with refined selection procedures that promote convergence to the Pareto front while preserving solution diversity. The enhanced MOPSO provides an improved exploration–exploitation solution for addressing large-scale, limited CLSC problems with various sustainability objectives by revising the global and individual best positions via non-dominated sorting.

In complex multi-objective problems, including sustainable CLSC networks under fuzzy uncertainty, metaheuristic algorithms are preferred over exact methods due to their flexibility and ability to escape local optima in large, nonlinear search spaces.

- *MOPSO − *It makes upon the concepts of PSO, where a swarm of particles explores the search space to discover optimal solutions. MOPSO enhances this methodology by simultaneously addressing multiple targets, aiming to identify a set of solutions that are not dominated^36^. Its efficacy is especially remarkable in achieving accelerated convergence rates and improving solution quality, which is approximated to that of alternative algorithms.
- *NSGA-II − *The present study utilizes NSGA-II, introduced^37^ to address the computational settings. This approach is efficient for multi-objective combinatorial optimization problems and their numerous variants, including assignment, distribution, and scheduling issues. Moreover, since it is a well-established algorithm, this overview does not include specifics regarding its steps and computations. It uses evolutionary principles selection, crossover, and mutation for generating a wide variety of Pareto-optimal solutions. It utilizes non-dominated sorted and overcrowding distance to preserve variety and enhance convergence towards the real Pareto front.

**Fig. 3:**
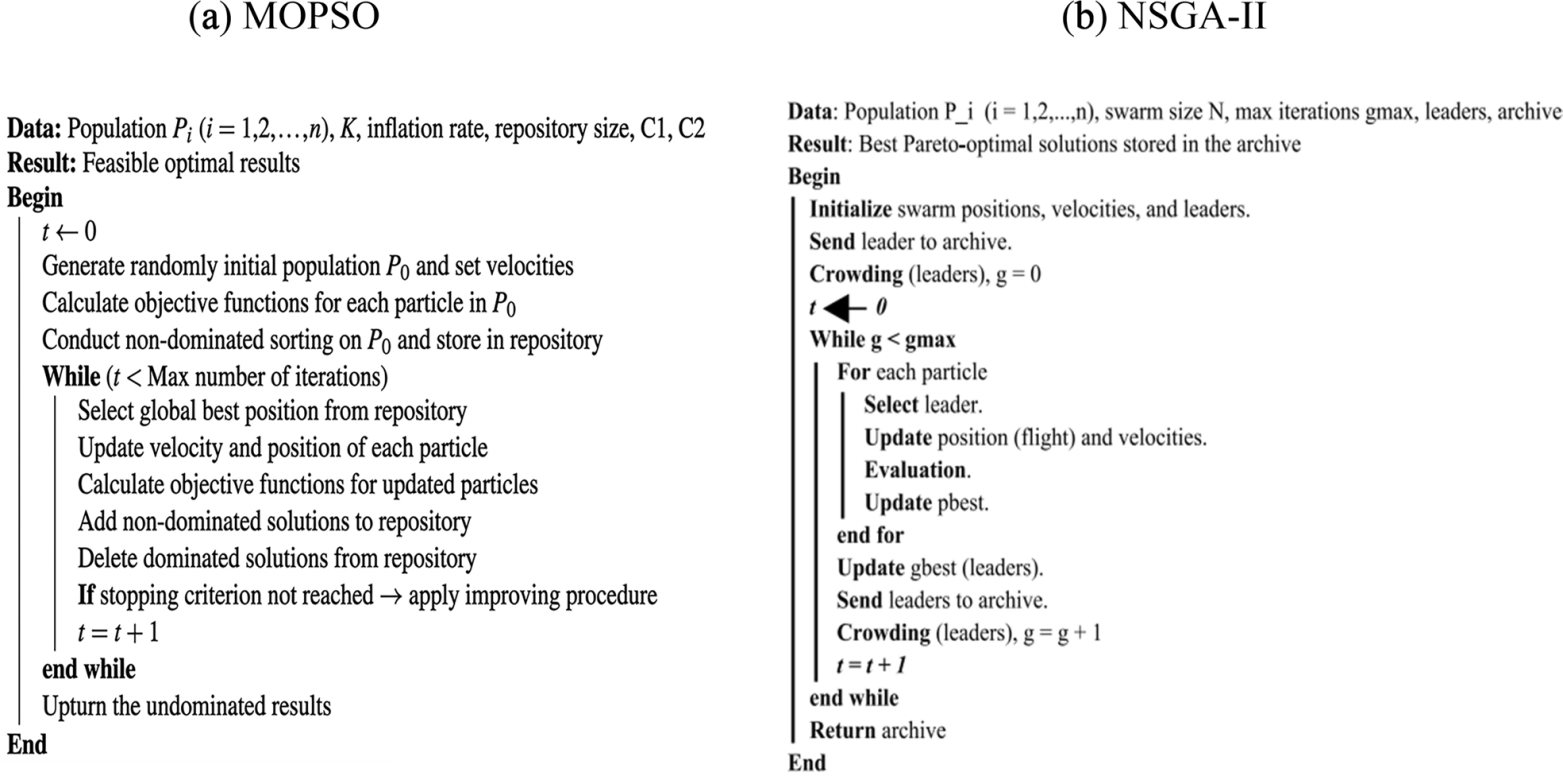
Pseudo-code of the algorithms.

To preserve conciseness and guarantee clarity in the paper, algorithm pseudo-codes are presented in Fig. 3. The algorithms commence the optimization process with a collection of initial feasible solutions produced during an organized setup phase.

## Case study and experimental results

Saba Battery Company, one of the largest and most reputable battery manufacturers in the Middle East, has over 40 years of experience in producing lead-acid automotive batteries ranging from 36 to 200 Ah. The company operates multiple production lines and business units, covering the full battery life cycle from raw material processing to final product testing and distribution. It utilizes advanced European technologies and has implemented dedicated systems for recycling, lead recovery, and environmental compliance. With an extensive distribution and reverse logistics network, Saba Battery supports the efficient recycling and disposal of end-of-life batteries. This comprehensive and sustainability-driven infrastructure makes it an ideal candidate for implementing the proposed CLSC optimization model.

The next part shows experimental results of evaluating and benchmarking the efficacy of the suggested hybrid metaheuristic framework, which combines NSGA-II and MOPSO for addressing the fuzzy multi-objective CLSC framework within the context of the Saba Battery Company case. A collection of mathematical test samples of diverse sizes was completed using operational data and parameters sourced from industry records and publicly accessible materials to facilitate realistic evaluation. The test problems are addressed utilizing both the suggested metaheuristic algorithms and an exact technique, specifically, the CPLEX solver developed by GAMS. Due to computational constraints, the CPLEX solver is limited to managing only small-scale problem samples within acceptable timeframes, permitting a comparative analysis of exact and metaheuristic solutions alone at this level. CPLEX was implemented through GAMS 42, while the metaheuristic algorithms were developed and executed in Python. The values ​​for each of the parameters are presented in Table 2.

**Table 2:** Results of optimization parameters and level settings in MOPSO.

Parameter	Values		
	Level 1	Level 2	Level 3
Iteration limit (Max-iteration)	100	200	300
Number of population (Npop)	50	100	150
Self-learning coefficient (C1)	1.5	2	2.5
Overall learning rate (C2)	1.5	2	2.5
Inflation rate	0.05	0.1	0.15
Repository size	10	15	20
Proportion of mutations (Pm)	0.1	0.2	0.3

The Taguchi L27 approach is an orthogonal experimental methodology that facilitates the systematic exploration of many variables at three levels while utilizing a minimized number of experiments. The L27 orthogonal array requires only 27 experimental runs to assess the main effects of up to 13 factors, making it an effective alternative to complete factorial experiments. This study uses the Taguchi L27 design to optimize the critical parameters of the proposed metaheuristic algorithms, facilitating an efficient and systematic assessment of parameter impacts on solution quality and convergence behavior while markedly reducing computational effort. Then, with Taguchi L27 design, different experiments were created (Table 3) and MOPSO was implemented for each of them.

**Table 3:** Variable response values in the Taguchi technique for MOPSO.

Run order	Max-iteration	Npop	C1	C2	Inflation rate	Repository size	Pm	MOPSO response
1	1	1	1	1	1	1	1	44,114,214
2	1	1	1	1	2	2	2	52,814,982
3	1	1	1	1	3	3	3	52,914,382
4	1	2	2	2	1	1	1	33,029,492
5	1	2	2	2	2	2	2	32,516,100
6	1	2	2	2	3	3	3	33,242,752
7	1	3	3	3	1	1	1	36,114,110
8	1	3	3	3	2	2	2	23,504,278
9	1	3	3	3	3	3	3	23,067,566
10	2	1	2	3	1	2	3	35,506,176
11	2	1	2	3	2	3	1	35,513,686
12	2	1	2	3	3	1	2	36,969,604
13	2	2	3	1	1	2	3	23,717,356
14	2	2	3	1	2	3	1	22,806,992
15	2	2	3	1	3	1	2	27,527,160
16	2	3	1	2	1	2	3	31,953,734
17	2	3	1	2	2	3	1	29,253,330
18	2	3	1	2	3	1	2	29,823,430
19	3	1	3	2	1	3	2	33,842,190
20	3	1	3	2	2	1	3	29,391,520
21	3	1	3	2	3	2	1	24,494,478
22	3	2	1	3	1	3	2	32,988,765
23	3	2	1	3	2	1	3	28,746,310
24	3	2	1	3	3	2	1	23,951,842
25	3	3	2	1	1	3	2	31,784,420
26	3	3	2	1	2	1	3	27,863,290
27	3	3	2	1	3	2	1	24,474,248

**Fig. 4:**
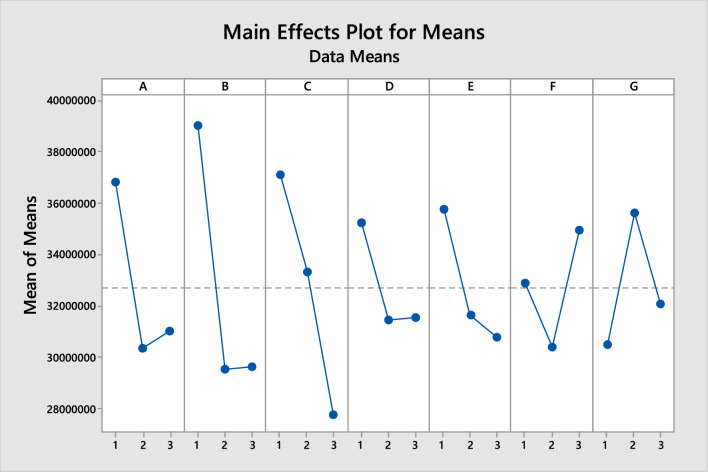
Analytical visualizations of MOPSO variables utilizing the Taguchi technique.

In summary, the results supplied to MINITAB program provide the S/N ratios comparing the two algorithms. Figure 4 shows the main effects of MOPSO control parameters (A–G) on the mean objective value using the Taguchi method. The plots reveal how different parameter levels influence solution quality, with steeper trends indicating more influential parameters and supporting efficient parameter tuning.

**Fig. 5:**
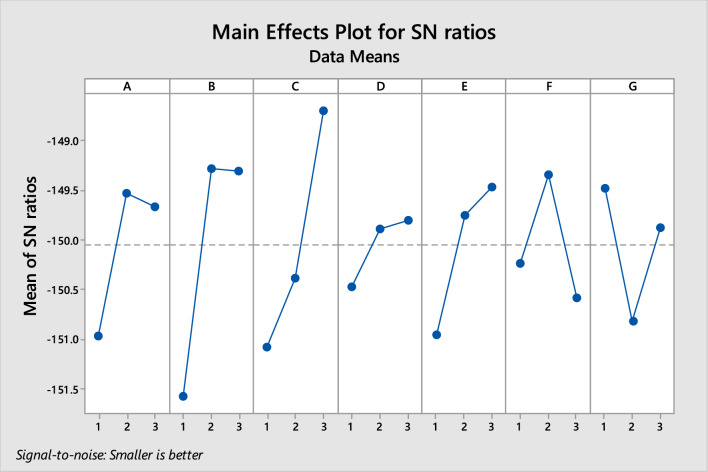
Analytical visualizations of NSGA-II variables utilizing the Taguchi technique.

Figure 5 indicates the principal effects plot for NSGA-II settings as a function of the signal-to-noise (S/N) ratio, under the "smaller-is-better" criteria. The S/N analysis emphasizes parameter robustness by identifying factor levels that reduce performance variability caused by uncertainty. Parameters with greater S/N variance across levels exhibit greater sensitivity and are therefore crucial for algorithm stability and convergence behavior.

Now, based on the output presented in the above diagram, the optimal value for each parameter is established, and additional instances are conducted using these parameter values of the method. Table 4 represents the ideal values of the variables.

**Table 4:** Optimal value of the variables in MOPSO.

Parameters	Optimal values
Iteration limit (Max-iteration)	100
Number of population (Npop)	50
Self-learning coefficient (C1)	1.5
Overall learning rate (C2)	1.5
Inflation rate	0.05
Repository size	20
Proportion of mutations (Pm)	0.2

### Comparison results

To compare the algorithms based on the studied indicators, 15 problem instances in different dimensions were generated. These examples are randomly generated and include all dimensions of the problem (i.e. small, medium, and large dimensions).

Table 5 provides a comparative assessment of the performance of NSGA-II, MOPSO, and CPLEX among small (S), medium (M), and large (L) scale problems. Table 5 presents the mean, coefficient of variation (CV), and standard deviation (Std.) for each algorithm over the specified test problem sizes. For small-scale problems (S1 to S5), both NSGA-II and MOPSO exhibit comparable performance, featuring low Std. and CV, signifying consistent outcomes. Table 5 demonstrates that CPLEX is constrained to small-scale instances of the approach, as it is unable to resolve medium-scale problems inside the 3500-s time constraint, underlining its increasing limitations.

**Table 5:** Performance comparison of NSGA-II, MOPSO, and CPLEX.

Test problems	NSGA-II mean	NSGA-II Std	NSGA-II CV	MOPSO mean	MOPSO Std	MOPSO CV	CPLEX mean
S1	513,651	40,572	7.90	343,636	30,056	8.75	499,494
S2	350,747	35,984	10.26	380,939	39,623	10.40	405,194
S3	531,370	33,214	6.25	336,030	26,284	7.82	481,169
S4	501,087	20,347	4.06	475,504	33,121	6.97	464,928
S5	487,947	34,227	7.01	512,821	40,740	7.94	420,657
M1	370,379	44,240	11.94	396,253	48,825	9.92	353,851
M2	474,025	24,010	5.07	564,926	23,772	4.21	396,820
M3	455,220	21,230	4.66	500,176	38,716	7.74	445,698
M4	328,608	25,320	7.71	427,214	46,705	10.93	486,438
M5	523,099	37,578	7.18	584,179	38,378	6.57	421,229
L1	501,087	20,347	4.06	475,504	33,121	6.97	464,928
L2	487,947	34,227	7.01	512,821	40,740	7.94	420,657
L3	328,608	25,320	7.71	427,214	46,705	10.93	486,438
L4	523,099	37,578	7.18	584,179	38,378	6.57	421,229
L5	501,087	20,347	4.06	475,504	33,121	6.97	464,928

**Fig. 6:**
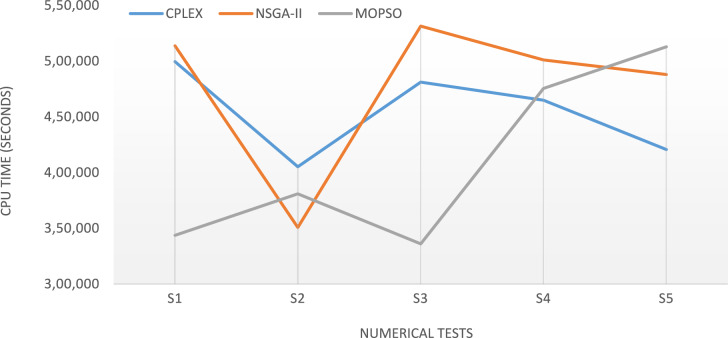
Computation time for NSGA-II, MOPSO, and CPLEX on small-size instances.

Figure 6 illustrates the computation time (in seconds) for NSGA-II, MOPSO, and CPLEX on small-scale instances (S1 to S5). NSGA-II and MOPSO consistently provide reliable performance with minimal computation times across all problems. CPLEX demonstrates markedly increased computation times, particularly for the more intricate test problems (S4 and S5), where its efficiency diminishes.

**Fig. 7:**
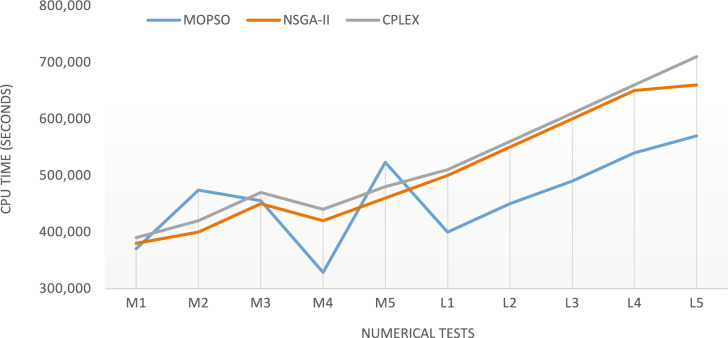
Computation time for NSGA-II, MOPSO, and CPLEX on medium and large-scale problems.

Figure 7 presents the computation time (in seconds) required by NSGA-II, MOPSO, and CPLEX across both medium (M1 to M5) and large (L1 to L5) scale problems. The plot indicates that CPLEX has significantly higher computation times compared to the metaheuristic algorithms (NSGA-II and MOPSO), especially as the problem size increases (from M to L). While NSGA-II and MOPSO indicate relatively stable growth in computation time, CPLEX increases sharply, reflecting its scalability limitations for larger problems. This suggests that for medium to large-scale problems, NSGA-II and MOPSO are much superior regarding computing time than CPLEX.

**Fig. 8:**
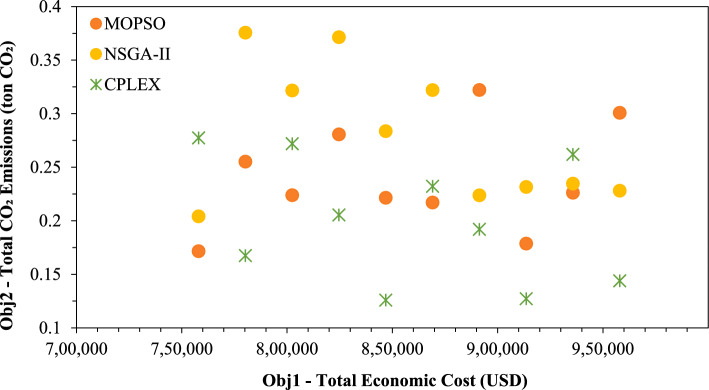
Pareto front derived from NSGA-II and MOPSO for sample problem S5 (small-scale problem).

The Pareto front in Fig. 8 for sample problem S5 compares the performance of MOPSO, NSGA-II, and CPLEX over two objective functions (Obj1 and Obj2). Objective 1 represents the total network cost including facility opening, transportation, processing, and recovery costs. Objective 2 represents the total carbon emissions generated from production, transportation, and recycling activities across the closed-loop supply chain. Consistent with the findings for test problem S4, both algorithms converge towards optimal values for the second objective (Obj2), with MOPSO and NSGA-II demonstrating superior performance in Obj1. Nonetheless, the two metaheuristics indicate significant differences in their approaches to balancing the objectives. MOPSO and NSGA-II achieve optimal solutions more rapidly than CPLEX, underscoring their efficiency in traversing the trade-off landscape. Although CPLEX can achieve optimal solutions, its computing time and convergence speed are inadequate to those of MOPSO and NSGA-II, which provide a superior balance between exploration and exploitation.

**Fig. 9:**
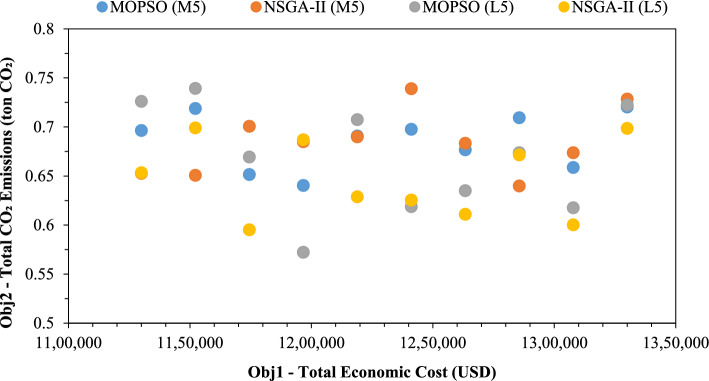
Pareto front derived using NSGA-II and MOPSO for test problems M5 and L5 (medium and large instances).

In Fig. 9, the Pareto front for M5 and L5 compares the performance of MOPSO and NSGA-II over two objective functions (Obj1 and Obj2). Consistent with the outcomes in M1, both MOPSO and NSGA-II indicate comparable performance in Obj2, with NSGA-II displaying somewhat superior outcomes in Obj1. Lower values of Objective 1 indicate improved economic efficiency, while lower values of Objective 2 indicate improved environmental sustainability of the battery closed-loop supply chain network. Nonetheless, NSGA-II exhibits better performance than MOPSO on the first goal (Obj1). As depicted in Fig. 9, the Pareto solutions derived from NSGA-II consistently yield lower Obj1 values, with minimum costs of approximately 1.15 × 10⁶ for NSGA-II compared to approximately 1.20 × 10⁶ for MOPSO under similar conditions, indicating an enhancement of roughly 4–5%. Furthermore, NSGA-II produces a more compact, uniformly distributed Pareto front. NSGA-II produces a greater density of non-dominated solutions within the Obj1 interval of 1.15–1.30 × 10⁶, while sustaining competitive Obj2 values (about 0.60–0.74); in contrast, MOPSO solutions display broader dispersion and a reduced number of dominant points in this area. This indicates that NSGA-II achieves a more efficient balance between exploration and exploitation, yielding more stable, well-distributed trade-off solutions. In contrast, MOPSO produces fewer Pareto solutions in this region and exhibits wider gaps between adjacent solutions, indicating weaker coverage of intermediate trade-offs. This demonstrates that NSGA-II provides a broader and more informative spectrum of trade-off solutions, offering decision-makers greater flexibility in balancing economic and secondary objectives.

Then, both algorithms are implemented with the optimal values ​​of their parameters. The values ​​of the desired indicators are calculated for each of the 10 examined examples. The performance indicators used to assess the efficiency and quality of solutions in multi-objective optimization problems are the mean ideal distance (MID):

71 $$MID = \frac{1}{N}\sum\nolimits_{{i = 1}}^{N} {\sqrt {\sum\nolimits_{{j = 1}}^{M} {\left( {f_{{ij}} - f_{{ij*}} } \right)} ^{2} } }$$

maximum distance (MD):

72 $$MD = \max \left( {\sqrt {\sum\nolimits_{{j = 1}}^{M} {\left( {f_{{ij}} - f_{{ij*}} } \right)^{2} } } } \right)$$

spread measure (SM):

73 $$SM = \frac{1}{{N - 1}}\sum\nolimits_{{i = 1}}^{{N - 1}} {\left| {f_{{i + 1}} - f_{i} } \right|}$$

number of Pareto solutions (NPS):

74 $$NPS = \sum\nolimits_{{i = 1}}^{N} {1(f_{i} )}$$

and rate of achievement of objectives (RAS):

75 $$RAS = \frac{1}{M}\sum\nolimits_{{j = 1}}^{M} {\frac{{f_{j}^{*} - f_{{\min }} }}{{f_{{\max }} - f_{{\min }} }}}$$

These metrics together provide a comprehensive assessment of the effectiveness of multi-objective optimization algorithms by evaluating both convergence (the proximity of solutions to the ideal) and diversity (the distribution of solutions across the Pareto front). Table 6 summarizes these results for NSGA-II.

**Table 6:** Output of NSGA-II for 10 examples.

Examples	MID	MD	SM	NPS	RAS	Spacing
1	69,479,059.9	19,478,628.7	0	50	25,873.3	178,501.0
2	88,657,879.1	27,071,626.2	0	50	112,620.1	155,934.9
3	93,294,193.1	3,669,143.9	44	6	438,553.4	796,515.5
4	175,315,303.5	7,579,387.7	46	4	1,895,455.5	680,406.2
5	168,357,764.0	3,583,376.6	44	6	0	677,397.2
6	66,975,208.2	6,120,874.9	40	10	432,362.4	414,292.6
7	222,976,934.0	3,302,833.6	37	13	254,819.6	212,999.6
8	418,741,560.9	3,308,433.3	44	6	552,457.0	180,297.5
9	665,192,553.3	4,150,407.6	42	8	266,287.2	462,310.5
10	1,396,720,286.8	6,275,471.0	43	7	0	548,444.9
Average	336,571,074.3	8,454,018.4	34	16	397,842.9	430,710.0

The results for 10 examples implemented by MOPSO are summarized in Table 7.

**Table 7:** MOPSO output solved for 10 examples.

Examples	MID	MD	SM	NPS	RAS	Spacing
1	46,876,560.7	59,696,763.2	85	15	838,060.7	2,437,252.7
2	61,102,089.2	23,242,381.6	86	14	649,822.2	2,458,350.2
3	80,961,799.3	0	87	13	0	0
4	139,484,526.3	24,635.6	97	3	0	12,361.4
5	106,607,694.0	8,381.8	98	2	0	0
6	56,855,730.6	19,547,250.8	85	15	1,236,107.5	570,653.7
7	82,250.6	82,250.6	95	5	16,787.8	13,740.7
8	260,508,067.0	35,203.4	98	2	0	0
9	716,904,029.8	0	88	12	0	0
10	1,199,394,860.0	53,302,013.0	46	4	0	219,735.0
Average	291,322,338.5	15,593,888.0	86.5	8.5	274,077.8	571,209.4

**Fig. 10:**
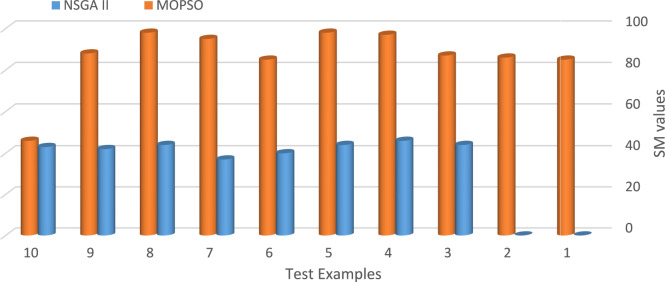
Results of the comparative analysis of the algorithms using the SM index.

Figure 10 (SM index) indicates the diversity of solutions as measured by the spread metric. Table 6 demonstrates that NSGA-II attains a significantly lower average SM value (34) compared to MOPSO (86.5; Table 7). Given that lower SM values signify superior distribution uniformity, Fig. 10 substantiates that NSGA-II generates more uniformly distributed Pareto solutions across the majority of test instances.

**Fig. 11:**
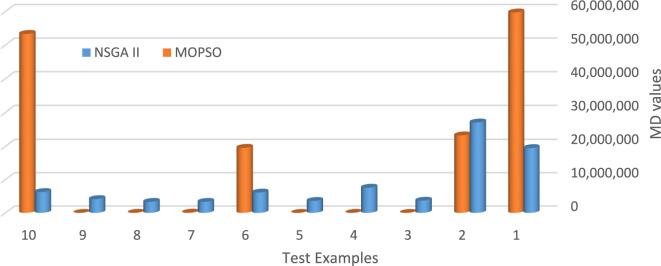
Results of the comparative analysis of the algorithms using the MD index.

Figure 11 (MD index) indicates the peak in solution diversity. MOPSO achieves a superior average MD value (15,593,888.0) compared to NSGA-II (8,454,018.4), indicating a broader solution space. This indicates that MOPSO prioritizes extensive exploration; however, this benefit does not inherently result in improved uniformity or dominance, as evidenced by other measures.

**Fig. 12:**
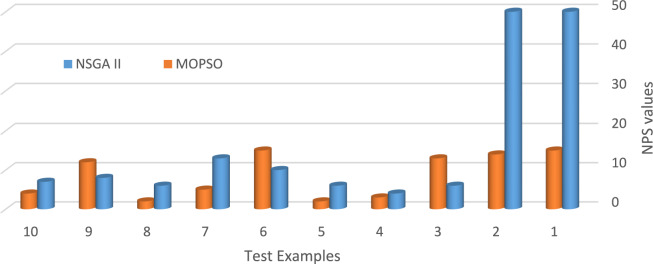
Results of the comparative analysis of the algorithms using the NPS index.

Figure 12 (NPS index) evaluates the number of Pareto-optimal solutions obtained. NSGA-II clearly outperforms MOPSO with an average NPS of 16, nearly double that of MOPSO (8.5). This demonstrates NSGA-II’s superior ability to identify and preserve non-dominated solutions, which is particularly important for decision-makers who require multiple trade-off options.

**Fig. 13:**
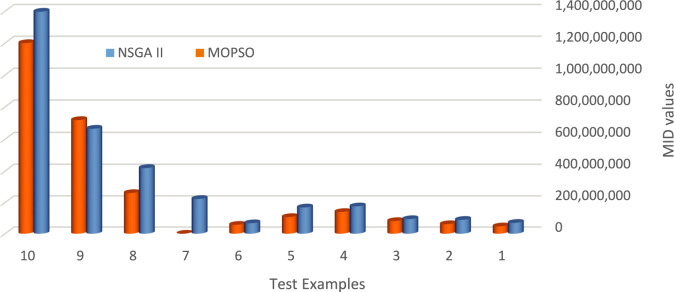
Results of the comparative analysis of the algorithms using the MID index.

Figure 13 (MID index) demonstrates convergence towards the optimal solution. Tables 6 and 7 indicate that MOPSO achieves lower average MID values (291, 322, 338.5) than NSGA-II (336, 571, 074.3), indicating superior convergence. This elucidates the diminished MID bars noted for MOPSO.

**Fig. 14:**
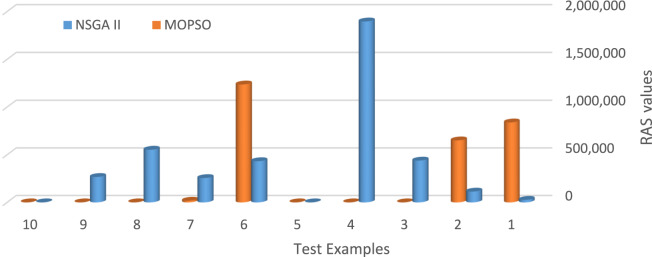
Results of the comparative analysis of the algorithms using the RAS index.

Figure 14 (RAS index) evaluates the simultaneous attainment of various objectives. NSGA-II attains a superior average RAS value (397,842.9) compared to MOPSO (274,077.8), signifying a fairer fulfillment of financial, environmental, and social objectives. Thus, Fig. 14 supports the assertion that NSGA-II indicates superior performance in multi-objective coordination.

**Fig. 15:**
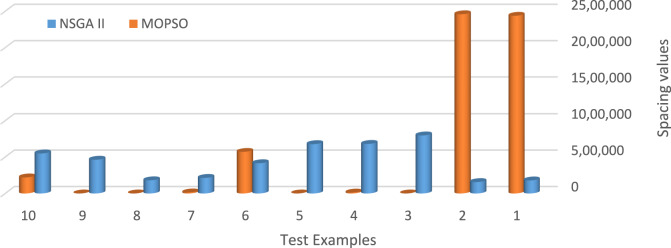
Results of the comparative analysis of the algorithms using the spacing index.

Figure 15 (spacing index) evaluates the consistency of intervals between consecutive Pareto solutions. NSGA-II demonstrates a markedly lower average spacing (430,710.0) compared to MOPSO (571,209.4), hence establishing a more uniform and stable Pareto front.

The comparative enhancement of the suggested MOPSO methodology over NSGA-II (or CPLEX baseline) is calculated as:

76 $${\mathrm{Im}} provment(\% ) = \frac{{Z_{base} - Z_{proposed} }}{{Z_{base} }} \times 100$$

where *Z* denotes the corresponding objective (total cost, total CO₂ emissions, or social index).

where ${Z_{base}}$​ and ${Z_{proposed}}$ represent the objective-function values obtained by the baseline algorithm (NSGA-II) and the proposed algorithm (MOPSO), respectively.

Table 8 represents a comparative evaluation of the sustainability performance of NSGA-II and MOPSO across the financial, environmental, and social factors. The findings demonstrate that MOPSO reduces overall system cost from 2 × 10⁶ to 1.87 × 10⁶, representing a 6.3% reduction, while total CO₂ emissions decrease by 8.1%. The social performance measure rises from 0.75 to 0.81, indicating a 12.5% enhancement in social benefit. The quantitative results indicate that the proposed MOPSO system achieves significant improvements across all three sustainability objectives.

**Table 8:** Comparative performance improvement of NSGA-II relative to MOPSO based on multi-objective evaluation metrics.

Metric	NSGA-II (base)	MOPSO (proposed)	Improvement (%)
Total cost	2 × 10^6^	1.87 × 10^6^	- 6.3% (Decrease)
CO_2_	20	18.5	- 8.1% (Decrease)
Social (0–1)	0.75	0.81	+ 12.5% (Increase)

### Sensitivity analysis

The results of solving the developed model based on the adaptive evolutionary computation (AEC) are presented by considering diverse values ​​of the parameter *λ* (i.e. the optimism–pessimism) as well as various uncertainty levels for the confidence level parameters *α*, *β*, and *γ*.

**Fig. 16:**
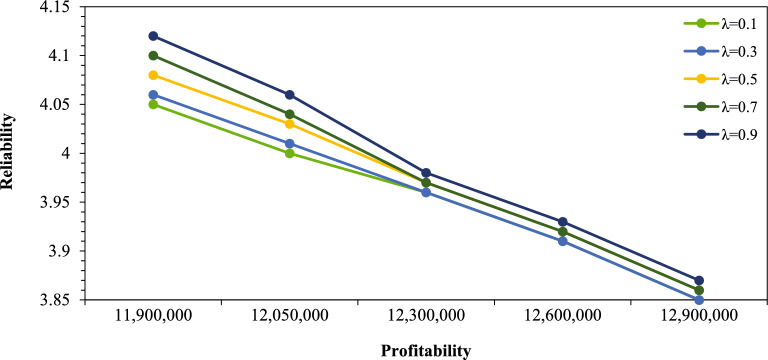
Overview of AEC results in profitability vs. reliability.

Figure 16 presents the trade-off between profitability and dependability across varying optimism–pessimism levels by utilizing the AEC approach. As *λ* increases, indicating a more optimistic decision-making approach, the solutions exhibit enhanced reliability at marginally reduced profitability levels. Figure 16 illustrates a persistent negative correlation; i.e., increasing profitability is associated with a decrease in system reliability. This pattern validates the model’s responsiveness to risk preferences, emphasizing the need for decision-makers to hit a balance between economic benefits and reliable SC performance amid uncertainty.

**Fig. 17:**
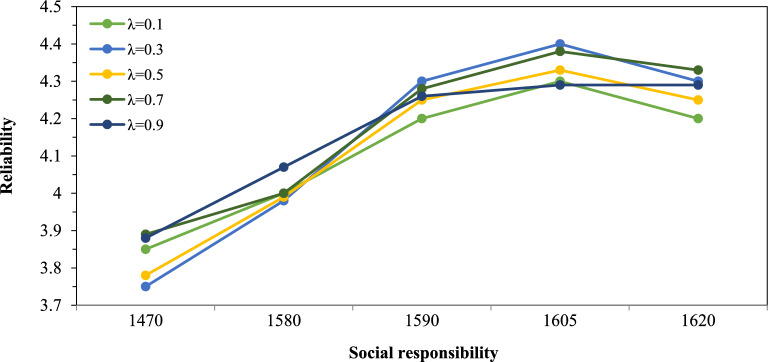
Overview of the AEC results in social responsibility vs. reliability.

Figure 17 presents the correlation between social responsibility and reliability across different levels of the optimism–pessimism *λ*. It highlights the system’s behavioral changes in response to uncertainty. The trend demonstrates that reliability often rises with social responsibility to a certain extent, beyond which it experiences a modest decrease, indicating a non-linear trade-off. Increased *λ* (i.e. more optimistic decision-making) often leads to enhanced reliability, underscoring that risk-tolerant techniques yield superior system performance in terms of dependability. This outcome corroborates the model’s efficacy in accurately representing realistic uncertainty management in sustainable CLSC planning.

**Fig. 18:**
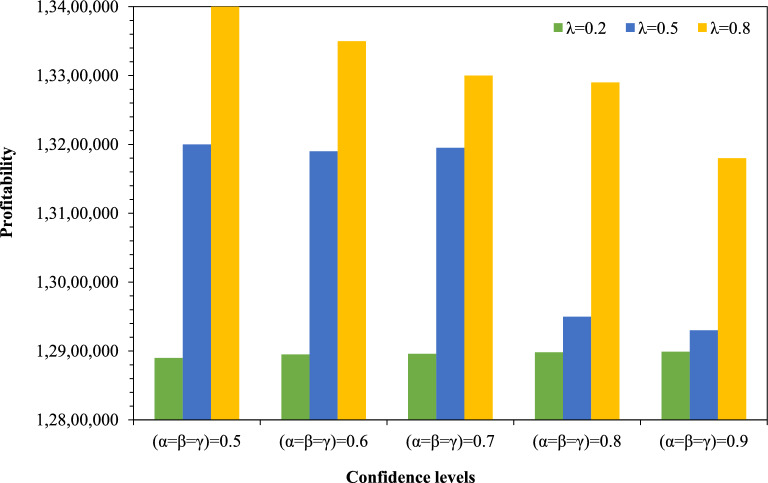
Impact of concurrent modifications in confidence level variables on profitability.

SC profitability is used to examine the effect of simultaneous changes in the parameters *α*, *β*, and *γ* (confidence levels) and the optimism–pessimism parameter. These changes are shown in Fig. 18. As can be seen, reducing the level of uncertainty and increasing the optimism–pessimism *λ* increases the value of SC profitability. This conclusion is based on the fact that the manager’s risk tolerance level has increased, thereby enhancing the profitability of SC. On the other hand, increasing the level of confidence, decreasing the parameter *λ*, and reducing the manager’s risk tolerance level all decrease the profitability of SC. In other words, by increasing the confidence levels *α*, *β*, and *γ*, the reasonable area of ​​the solution becomes smaller, and worse values ​​are reported as the solution to the problem.

### Managerial implications

Developing and designing a sustainable CLSC network is complex due to the conflicting objectives and uncertainties involved in its structure. The principle of sustainable development is essential at many tiers of the CLSC, according to its increasing significance in modern society. Consequently, an effective technique must be established to tackle the challenges identified in the research conducted. A novel fuzzy optimization approach, extending prior models in the literature, was initially constructed to establish the primary component of the methodology. The suggested model was subsequently validated, and its applicability was assessed through a real-world case study supported by several sensitivity analyses. From a managerial point of view, it was demonstrated that a 10% increase in manufacturing capacities at each level leads to a significant reduction in bottleneck requests.

The approach suggests how executives might mitigate bottleneck requests by developing improved manufacturing capacities or alternative sectors, thereby maximizing profit. Conversely, considering additional assets or their availability is essential, especially in volatile real-world scenarios where SC’s flexibility is optimized for sustainability and uncertainty. Furthermore, managers should prioritize outsourcing strategies, particularly during peak demand seasons, which is strongly advised. From the standpoint of uncertainty, managers can address all needs by selecting the maximum confidence level and minimum threat value.

## Conclusion

This study aimed to develop a multi-objective CLSC network model under uncertainty. The model was developed to simultaneously reduce overall SC costs, enhance customer satisfaction, and decrease transportation-related emissions, thereby achieving objectives. A multi-objective MILP model was developed to reduce the costs and simultaneously maximize total profitability and enhance customer satisfaction across the CLSC.

A comprehensive SC network model specific to the battery recycling sector was developed, encompassing both forward and reverse operational actions. The model was resolved to utilize the GAMS platform for minor examples to ascertain feasibility and performance. Two sophisticated metaheuristic algorithms NSGA-II and MOPSO were utilized for large problem sets. The comparison findings suggested that MOPSO outperformed NSGA-II in the majority of evaluation criteria, yielding solutions that were more closely connected with the optimal front. In the present study, the battery closed-loop supply chain model exhibits a structured but relatively smooth objective landscape after defuzzification. Under these conditions, the swarm-based memory-sharing mechanism of MOPSO enhanced convergence speed and objective refinement, resulting in lower MID values and improved objective outcomes. However, NSGA-II demonstrated stronger diversity performance (higher NPS and improved spacing indicators), consistent with its crowding-distance-based preservation mechanism.

Overall, MOPSO outperformed NSGA-II across all objectives, delivering 6.3% lower cost, 8.1% lower emissions, and 12.5% higher social benefit, demonstrating improved convergence performance and practical decision-making advantages under the evaluated computational settings for sustainable closed-loop supply-chain design.

Future research should enhance the model’s robustness by integrating uncertainty across additional parameters such as costs, transportation, and return rates, potentially using machine learning and predictive analytics for dynamic demand modeling. Incorporating time-window constraints can improve the representation of perishability and delivery precision, while explicitly accounting for waste by-products and non-recyclable residues will strengthen the model’s environmental sustainability.

## Supplementary Information

Supplementary Information.

## Data Availability

The data findings of this study are available from the corresponding author upon reasonable request.
