# Supplementary material for: Sustainable closed-loop supply chain network design under uncertainty using a fuzzy multi-objective optimization framework for the battery industry
Source: Sci Rep. 2026 Apr 16;16:17748. doi: 10.1038/s41598-026-47477-8 (PMC13246781; doi:10.1038/s41598-026-47477-8)
Supplement: Supplementary file 1 — Supplementary Information. [file 41598_2026_47477_MOESM1_ESM.docx]

Supplementary file

Appendix-A: Notations

***Indices:***

| *s* | Index of suppliers |
| --- | --- |
| *m* | Index of pants |
| *d* | Index of distribution centers |
| *r* | Index of disposal/burial centers |
| *c* | Index of customers |
| *v* | Index of purchased vehicles |
| *w* | Index of rented vehicles |
| *i,j* | Index of candidate sites for *m,d* |
| *l* | Index of recycling center |
| *k* | Index of raw materials |
| *p* | Index of products |
| *t* | Periods with fixed π length |

***Parameters:***

| ${C1}_{ksmv}$ | Cost of transportation of each unit of the raw materials *k* from supplier *s* to production center *m* with purchased vehicle *v* |
| --- | --- |
| ${C2}_{pmdv}$ | Cost of transportation of each unit of the final product *p* from production center *m* to distribution center *d* with purchased vehicle *v* |
| ${C3}_{pdcv}$ | Cost of transportation of final product center *p* from distribution center *d* with purchased vehicle *v* |
| ${C4}_{pdrv}$ | Cost of transportation of final product center *p* from distribution center *d* to disposal center *r* with purchased vehicle *v* |
| ${C5}_{pdlv}$ | Cost of transportation of final product center *p* from distribution center *d* to recycling center *l* with purchased vehicle *v* |
| ${C6}_{klsv}$ | Cost of transportation of raw material *k* from recycling center *l* to supply center *s* with purchased vehicle *v* |
| ${CC1}_{ksmv}$ | Cost of transportation of raw material *k* from production center *m* to supply center *s* with purchased vehicle *w* |
| ${CC2}_{pmdv}$ | Cost of transportation from end product *p* from production center *m* to distribution center *d* with purchased vehicle *v* |
| ${CC3}_{pdcv}$ | Cost of transportation from end product *p* from distribution center *d* to customer *c* with purchased vehicle *v* |
| ${CC4}_{pdrv}$ | Cost of transportation of final product center *p* from distribution center *d* to disposal center *r* with purchased vehicle *v* |
| $C{C5}_{pdlv}$ | Cost of transportation of final product center *p* from distribution center *d* to recycling center *l* with purchased vehicle *v* |
| ${CC6}_{klsv}$ | Cost of transportation of raw material *k* from recycling center *l* to supply center *s* with purchased vehicle *v* |
| ${h1}_{km}$ | Cost of warehousing on each raw material *k* in production center *m* |
| ${h2}_{pm}$ | Cost of warehousing on final product *p* in production center *m* |
| ${h3}_{pd}$ | Cost of warehousing on final product *p* in distribution center *d* |
| ${h4}_{pr}$ | Cost of disposal of product *p* in distribution center *r* |
| ${h5}_{pl}$ | Cost of recycling of product *p* in recycling center *l* |
| ${\pi1}_{pd}$ | Cost of shortage of final product *p* in distribution center *d* |
| ${\pi2}_{pc}$ | Cost of shortage of final product *p* in customer demand *c* |
| ${\pi3}_{km}$ | Cost of shortage of raw material *k* in production center *m* |
| ${f1}_{mi}$ | Fixed cost of starting production center *m* in place *i* |
| ${f2}_{dj}$ | Fixed cost of distribution center *d* in place *j* |
| ${f3}_{r}$ | Fixed cost of disposal and burial center *r* |
| ${f4}_{l}$ | Fixed cost of starting recycling center *l* |
| ${PT}_{pm}$ | Time required for product *p* in production center *m* |
| ${ST}_{pm}$ | Time required for starting product *p* in production center *m* |
| ${p1}_{ksm}$ | Cost of purchasing raw material *k* from supply center *s* by production center *m* |
| ${p2}_{pmd}$ | Cost of purchasing final product *p* from production center *m* by distribution center *d* |
| ${p3}_{pdc}$ | Profit of selling final product *p* from distribution center *d* by customer *c* |
| ${l1}_{sm}$ | Distance from supply center *s* to production center *m* |
| ${l2}_{md}$ | Distance from production center *m* to distribution center *d* |
| ${l3}_{dc}$ | Distance from distribution center *d* to customer center *c* |
| ${l4}_{dr}$ | Distance from distribution center *d* to disposal center *r* |
| ${l5}_{dl}$ | Distance from distribution center *d* to recycling center *l* |
| ${l6}_{ls}$ | Distance from distribution center *l* to supply center *s* |
| $e_{pm}$ | Cost of production *p* in production center *m* |
| $\tilde{TD}_{pct}$ | Fuzzy demand of customer *c* in period *t* from final product *p* |
| ${\alpha1}_{v}$ | Dispersion of CO_2_ from purchased vehicle *v* in empty state |
| ${\alpha2}_{v}$ | Dispersion of CO_2_ from purchased vehicle *v* in full state |
| ${\alpha\alpha1}_{w}$ | Dispersion of CO_2_ from rented vehicle *w* in empty state |
| ${\alpha\alpha2}_{w}$ | Dispersion of CO_2_ from rented vehicle *w* in full state |
| ${v1}_{v}$ | Weight capacity of purchased vehicle *v* |
| $v{v1}_{w}$ | Weight capacity of rented vehicle *w* |
| ${v2}_{m}$ | Weight capacity of production center *m* for warehousing raw material |
| ${v3}_{m}$ | Weight capacity for final product in production center *m* |
| ${v4}_{dp}$ | Weight capacity for final product *p* in distribution center *d* |
| ${g1}_{k}$ | Weight of each unit of raw material *k* |
| ${g2}_{p}$ | Weight of each unit of final product *p* |
| ${TT}_{t}$ | Total access time for production in period *t* |
| $R1$ | The number of centers for distribution for beginning |
| $R2$ | Number of disposal and burial centers |
| $R3$ | Number of production centers |
| ${R4}_{pk}$ | Required amount of raw material *k* in production of product *p* |
| $R5$ | Number of recycling centers |

***Decision-making variables:***

| ${P1}_{ksmtv}$ | Amount of supplied raw *k* materials from supplier *s* to production center *m* in period *t* by purchased vehicle *v* |
| --- | --- |
| ${P2}_{pmdtv}$ | Amount of supplied product *p* from production center *m* to distribution center *d* in period *t* by purchased vehicle *v* |
| ${q3}_{pdctv}$ | Amount of supplied product *p* from distribution center *d* to customer *c* in period *t* by purchased vehicle *v* |
| ${q4}_{pdrtv}$ | Amount of supplied product *p* from distribution center *d* to disposal center *r* in period *t* by purchased vehicle *v* |
| ${q5}_{pdltv}$ | Amount of supplied product *p* from distribution center *d* to recycling center *l* in period *t* by purchased vehicle *v* |
| ${q6}_{klstv}$ | Amount of supplied *p* from recycling center *l* to supplier *s* in period *t* by purchased vehicle *v* |
| ${qq1}_{ksmtw}$ | Amount of supplied raw *k* materials from supplier *s* to production center *m* in period *t* by rented vehicle *w* |
| ${qq2}_{pmdtw}$ | Amount of supplied product *p* from production center *m* to distribution center *d* in period *t* by rented vehicle *w* |
| ${qq3}_{pdctw}$ | Amount of supplied *p* from distribution center *d* to customer *c* in period *t* by rented vehicle *w* |
| ${qq4}_{pdrtw}$ | Amount of supplied product *p* from distribution center *d* to disposal center *r* in period *t* by rented vehicle *w* |
| ${qq5}_{pdltw}$ | Amount of supplied product *p* from distribution center *d* to recycling center *l* in period *t* by rented vehicle *w* |
| ${qq6}_{klstw}$ | Amount of raw product *k* from recycling center *i* to supplier *s* in period *t* by rented vehicle *w* |
| ${q4}_{pmt}$ | Amount of product *p* in factory *m* in period *t* |
| ${i1}_{kmt}$ | End period inventory of raw material *k* in production center *m* in period *t* |
| ${i2}_{pmt}$ | End period inventory of final product *p* in production center *m* in period *t* |
| ${i3}_{pdt}$ | End period inventory of final product *p* in distribution center *d* in period *t* |
| ${b1}_{kmt}$ | Shortage of raw material *k* in production center *m* in period *t* |
| ${b2}_{pdt}$ | Shortage of product *p* in production center *m* in period *t* |
| ${b3}_{pct}$ | Shortage of product *p* by customer demand *c m* in period *t* |
| ${x0}_{pmt}$ | If the production center *m* in period *t* produces *p* (it is one; otherwise, zero) |
| ${x1}_{mi}$ | If production center *m* is established in place *i* (it is one; otherwise, zero) |
| ${x2}_{dj}$ | If distribution center *d* is established in place *j* (it is one; otherwise, zero) |
| ${x3}_{r}$ | If disposal and burial center *r* is established (it is one; otherwise, zero) |
| ${x10}_{l}$ | If recycling center *l* is established (it is one; otherwise, zero) |
| ${x4}_{ksmtv}$ | If purchased raw material *k* from supply center *s* is transferred to production center *m* in period *t* by purchased vehicle *v* (it is one; otherwise, zero) |
| ${x5}_{pmdtv}$ | If product *p* from production center *m* is transferred to distribution center *d* in period *t* by purchased vehicle *v* (it is one; otherwise, zero) |
| ${xl6}_{pdctv}$ | If product *p* from distribution center *d* is transferred to customer *c* in period *t* by purchased vehicle *v* (it is one; otherwise, zero) |
| ${x7}_{pdrtv}$ | If product *p* from distribution center *d* is transferred to disposal and burial center *r* in period *t* by purchased vehicle *v* (it is one; otherwise, zero) |
| ${x8}_{pdItv}$ | If product *p* from distribution center *d* is transferred to recycling center *I* in period *t* by purchased vehicle *v* (it is one; otherwise, zero) |
| ${x9}_{klstv}$ | If the purchased *K* raw material from recycling center *l* is transferred to supplier *s* in period *t* by purchased vehicle *v* (it is one; otherwise, zero) |
| ${xx4}_{ksmtw}$ | If the purchased *k* raw material from supply center *s* is transferred to production center *m* in period *t* by purchased vehicle *v* (it is one; otherwise, zero) |
| ${xx5}_{pmdtw}$ | If product *p* from production center *m* is transferred to distribution center *d* in period *t* by rented vehicle *w* (it is one; otherwise, zero) |
| ${xx6}_{pdctw}$ | If product *p* from distribution center *d* is transferred to customer *c* in period *t* by rented vehicle *w* (it is one; otherwise, zero) |
| ${xx7}_{pdrtw}$ | If product *p* from distribution center *d* is transferred to disposal and burial center *r* in period *t* by rented vehicle *w* (it is one; otherwise, zero) |
| ${xx8}_{pditw}$ | If product *p* from distribution center *d* is transferred to recycling center *i* to the in-period *t* by rented vehicle *w* (it is one; otherwise, zero) |
| ${xx9}_{kIstw}$ | If the purchased *k* raw material from recycling center *l* is transferred to supplier *s* in period *t* by rented vehicle *w* (it is one; otherwise, zero) |
